# Transcriptional Regulation of Chemical Diversity in Aspergillus fumigatus by LaeA

**DOI:** 10.1371/journal.ppat.0030050

**Published:** 2007-04-13

**Authors:** Robyn M Perrin, Natalie D Fedorova, Jin Woo Bok, Robert A Cramer, Jennifer R Wortman, H. Stanley Kim, William C Nierman, Nancy P Keller

**Affiliations:** 1 Department of Plant Pathology, University of Wisconsin-Madison, Madison, Wisconsin, United States of America; 2 The Institute for Genomic Research, Rockville, Maryland, United States of America; 3 Department of Molecular Genetics and Microbiology, Duke University Medical Center, Durham, North Carolina, United States of America; 4 Department of Medicine, Korea University Medical College, Seoul, Korea; 5 Department of Biochemistry and Molecular Biology, The George Washington University School of Medicine, Washington, D. C., United States of America; David Geffen School of Medicine at the University of California Los Angeles, United States of America

## Abstract

Secondary metabolites, including toxins and melanins, have been implicated as virulence attributes in invasive aspergillosis. Although not definitively proved, this supposition is supported by the decreased virulence of an Aspergillus fumigatus strain, *ΔlaeA,* that is crippled in the production of numerous secondary metabolites. However, loss of a single LaeA-regulated toxin, gliotoxin, did not recapitulate the hypovirulent *ΔlaeA* pathotype, thus implicating other toxins whose production is governed by LaeA. Toward this end, a whole-genome comparison of the transcriptional profile of wild-type, *ΔlaeA,* and complemented control strains showed that genes in 13 of 22 secondary metabolite gene clusters, including several A. fumigatus–specific mycotoxin clusters, were expressed at significantly lower levels in the *ΔlaeA* mutant. LaeA influences the expression of at least 9.5% of the genome (943 of 9,626 genes in A. fumigatus) but positively controls expression of 20% to 40% of major classes of secondary metabolite biosynthesis genes such as nonribosomal peptide synthetases (NRPSs), polyketide synthases, and P450 monooxygenases. Tight regulation of NRPS-encoding genes was highlighted by quantitative real-time reverse-transcription PCR analysis. In addition, expression of a putative siderophore biosynthesis NRPS (NRPS2/*sidE*) was greatly reduced in the *ΔlaeA* mutant in comparison to controls under inducing iron-deficient conditions. Comparative genomic analysis showed that A. fumigatus secondary metabolite gene clusters constitute evolutionarily diverse regions that may be important for niche adaptation and virulence attributes. Our findings suggest that LaeA is a novel target for comprehensive modification of chemical diversity and pathogenicity.

## Author Summary

Patients with suppressed immune systems due to cancer treatments, HIV/AIDS, or organ transplantation are at high risk of infection from microbes. Some of the most deadly infections for such patients arise from a fungal pathogen, Aspergillus fumigatus. This species, like several of its close relatives, can produce an array of small chemical compounds that influences both the infection process and its environmental niche outside of the host. The genes dedicated to production of each compound are clustered adjacent to each other in the genome. One protein named LaeA is a master regulator of such clustered small molecule genes, and removal of the gene encoding LaeA cripples the organism's ability to infect. We conducted a genome-wide microarray experiment to identify small molecule gene clusters controlled by the presence of LaeA in A. fumigatus. In doing so, we identified actively expressed gene clusters critical for small molecule production and potentially involved in disease progression. These results also provide insight into evolutionary events shaping the organism's collection of chemical compounds.

## Introduction

Aspergillus fumigatus is a saprophytic filamentous fungus with no known sexual stage. Prolific production of asexual spores (conidia) and nearly ubiquitous distribution in the environment ensures constant host exposure to its spores, at a density of 1 to 100 conidia/m^−3^ [1]. The innate immune system enables spores to be eliminated from lung epithelial tissue with ease in immunocompetent vertebrates. However, immunocompromised individuals are at risk for pulmonary disease as a consequence of *A. fumigatus* infection. Of particular concern is invasive aspergillosis, which occurs when hyphal growth proliferates throughout pulmonary or other tissues. Invasive aspergillosis has an associated mortality rate ranging from 50% to 90% depending on the patient population [2]. As the number of immunocompromised patients has increased in recent decades due to immunosuppressive chemotherapy treatments, HIV/AIDS, and solid organ and bone marrow transplantation, the incidence of invasive aspergillosis has increased more than 4-fold in developed nations [2].

Several A. fumigatus secondary metabolites or natural products (e.g., conidial melanins and mycotoxins) have been implicated as affecting virulence [3–7]. However, the exact mechanisms by which many of these compounds might affect disease outcome are unknown, nor is it clear in most cases whether these factors play direct or indirect roles in pathogenicity. In contrast to most genes involved in primary metabolism, genes encoding secondary metabolite biosynthetic enzymes exist in contiguous clusters within the genome [8,9]. LaeA was originally identified as a transcriptional regulator of secondary metabolite gene clusters in Aspergillus nidulans and A. fumigatus [10,11], including gliotoxin in the latter. Gliotoxin has long been suggested to be a major virulence attribute in invasive aspergillosis [12–14]. However, whereas a *ΔlaeA* mutant shows reduced virulence in a mouse model of invasive aspergillosis [11], inactivation of gliotoxin biosynthesis alone does not [15–17]. Therefore, we reasoned that because LaeA is a transcriptional regulator, perhaps acting at a chromatin remodeling level [9,18], a microarray experiment comparing the transcriptomes of *ΔlaeA,* wild-type, and complemented *ΔlaeA* control strains would yield further insight into LaeA-mediated A. fumigatus virulence attributes. We uncovered an unprecedented view of LaeA global regulation of mycotoxin islands, nearly all found in nonsyntenic regions of the *Aspergillus* genome. Because secondary metabolite gene cluster regions are evolutionarily diverse and may affect virulence attributes, LaeA is a novel target for comprehensive modification of chemical diversity.

## Results/Discussion

### Microarray Data Generation and Quality Assessment

Transcriptional profiles of the wild-type, *ΔlaeA,* and *ΔlaeA* complemented strain were determined by comparisons of relative transcript levels between (1) *ΔlaeA* versus wild-type and (2) wild-type versus complemented control strain. All strains were grown under identical conditions (25 °C, liquid shaking culture, glucose minimal media, 60 h) for three biological replicates. The condition and time point were chosen on the basis of optimal production of secondary metabolites [10,11]. The comparison of *ΔlaeA* versus wild-type was used to determine gene expression patterns specific to the *ΔlaeA* mutant, while the wild-type versus complemented strain comparison was conducted as a control, because the difference between these two strains is the presence of an ectopic copy of a selectable marker for hygromycin resistance. The processed signal intensity ratios for the three *ΔlaeA* versus wild-type replicates were analyzed using the significance analysis of microarrays (SAM) method [19], as described in Materials and Methods. In total, 943 genes were significantly differentially expressed. Figure S1 shows a heat map of a subset of these loci, depicting normalized expression ratios for the three *ΔlaeA* versus wild-type experiments and the three wild-type versus complemented control experiments. The high quality of the data is indicated by the consistency of color between the replications and the relative lack of color in the control lanes.

### Patterns of Transcriptional Regulation

Of the 943 genes showing significant differences in expression between *ΔlaeA* and wild-type by SAM analysis, 415 showed increased expression in *ΔlaeA* and 528 showed decreased expression. Table 1 and Figure S2 indicate functional categories for these genes (defined as described by the Gene Ontology Consortium, http://www.geneontology.org). The most remarkable discovery was the near-global suppression of secondary metabolite gene expression in the *ΔlaeA* mutant. Nearly all (97%) of the secondary metabolite gene cluster loci showed decreased expression in *ΔlaeA,* with a mere three genes in this category showing increased expression in *ΔlaeA*. This was in contrast to all other functional categories, which showed substantial proportions of both increased and decreased expression in the mutant, possibly reflecting indirect effects due to loss of production of multiple metabolites. In addition to genes with unknown function (39%) and genes involved in secondary metabolism (11%), other major categories included genes encoding proteins involved in transmembrane transport (8%) and those involved in information processing (4%), and cell wall biogenesis (4%). Statistical analysis of the overrepresentation of different Gene Ontology categories and Pfam protein domains within the set of 943 differentially regulated genes is shown in Tables S1 and S2, respectively.

**Table 1:**
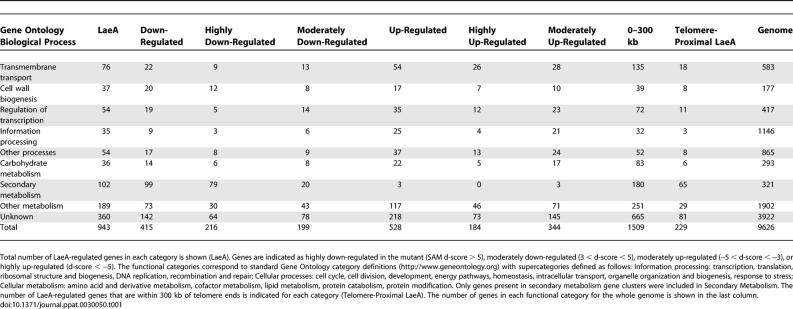
Gene Ontology Categories for Loci Regulated by LaeA

Interestingly, LaeA appeared to influence expression of a subset of species- and lineage-specific genes not strongly conserved with other fungal species. Only 18% and 44% of all genes significantly differentially expressed in the mutant have putative orthologs in Saccharomyces cerevisiae and *Neurospora crassa,* respectively, compared to an average of 33% and 58% of all A. fumigatus genes. Many, but not all, of these genes were classified as secondary metabolism genes. Moreover, there are about 120 differentially expressed genes; again, most, but not all, are present in secondary metabolism clusters (Table 2), which have no detectable orthologs in Aspergillus oryzae and A. nidulans. Considering this overwhelming tight and directed transcriptional control of secondary metabolite loci by LaeA, below we focus on such genes as possible members of the LaeA-regulated A. fumigatus pathogenicity arsenal.

**Table 2:**
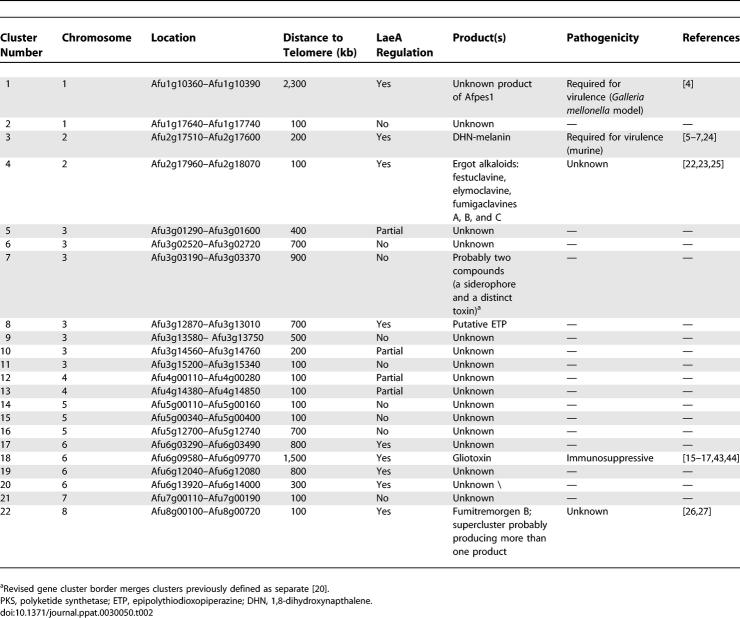
Summary of Secondary Metabolite Gene Cluster Function and Regulation by LaeA

### Regulation of Chemical Diversity by LaeA

**Figure 1:**
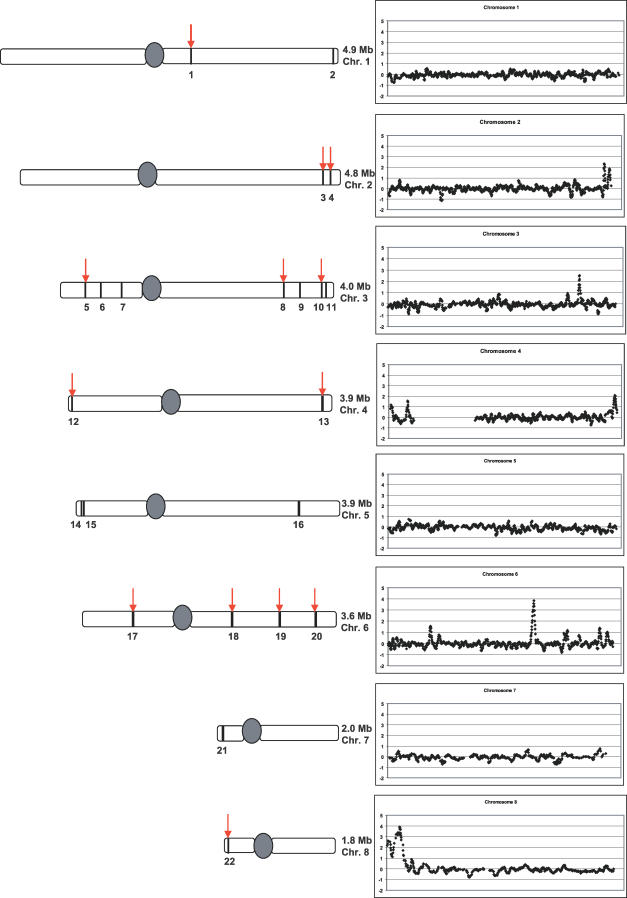
Positional Bias of LaeA-Regulated Gene Expression Left, chromosomes of A. fumigatus with secondary metabolite gene clusters indicated as bars and clusters numbered as indicated in Table 2. Arrows indicate clusters that are completely or partially regulated by LaeA. Graphs to the right of each chromosome show normalized expression ratio values of *ΔlaeA* versus wild-type plotted by chromosomal position. Each point represents the average value of ten contiguous loci. The gap on Chromosome 4 indicates a region of the genome not included in the microarray that contains the rRNA gene cluster. Positive values correspond to reduced expression in *ΔlaeA* and negative values correspond to increased expression in *ΔlaeA*.

Although initial genome analysis suggested the presence of 26 secondary metabolite gene clusters [20], subsequent analysis (G. Turner, N. D. Fedorova, V. Joardar, J. R. Wortman, and W. C. Nierman, unpublished data) has provided support for only 22 clusters. Of the 13 secondary metabolite gene clusters whose expression was influenced by LaeA in the condition used for microarray analysis, ten are particularly strongly affected, with a majority of genes within these clusters being significantly down-regulated in *ΔlaeA* as indicated by SAM. Three additional clusters have at least one gene encoding a critical enzyme such as a nonribosomal peptide synthetase (NRPS) or a polyketide synthase showing decreased expression in *ΔlaeA.* Additionally, 38% (23 of 71) of all P450 monooxygenases show differential expression in *ΔlaeA,* also associated with secondary metabolite biosynthesis and/or detoxification. Fifteen of these genes encoding P450 monooxygenases are found in secondary metabolite gene clusters. Table S3 gives normalized expression ratio values for all 22 gene clusters in *A. fumigatus.* Table 2 summarizes the current state of knowledge regarding function of LaeA-regulated secondary metabolite gene clusters. These include clusters dedicated to production of conidial melanins, fumitremorigens, gliotoxin, and ergot alkaloids such as festuclavine, elymoclavine, and fumigaclavines A, B, and C (Table 2) [4,17,21–27]. Figure 1 depicts the chromosomal landscape of those regions most strongly regulated by LaeA.

To confirm these microarray results, quantitative real-time reverse-transcription (RT)-PCR (QRT-PCR) was performed on one major class of secondary metabolite genes, those encoding NRPSs [28]. As indicated in Table 3, relative expression levels for NRPSs that showed differential expression between mutant and wild-type in the microarray study also were dramatically reduced upon QRT-PCR analysis. In all cases, complementation of the *laeA* defect restored NRPS gene expression to wild-type levels (Table 3).

**Table 3:**
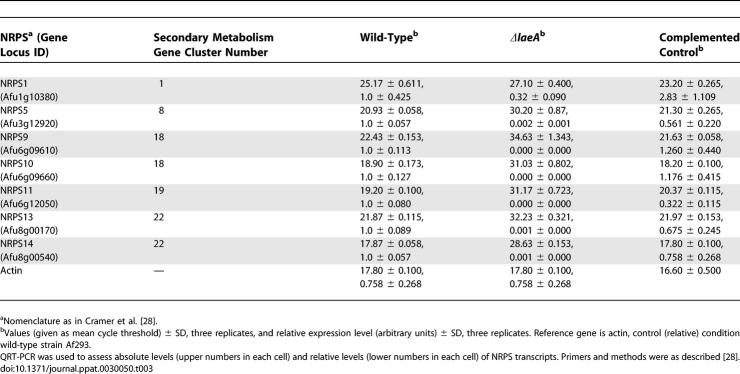
Real-Time RT-PCR Analysis of Select A. fumigatus NRPSs in Wild-Type, *ΔlaeA*, and Complemented Control Strains

Notably, because the microarray analysis determines only relative expression and not absolute levels of transcript, we could not conclude whether secondary metabolite clusters not showing differential expression in mutant versus wild-type were not affected by LaeA or were simply not induced under the growth condition used. To further examine these possibilities, we assessed the expression of a subset of NRPSs thought to encode siderophore-biosynthesizing enzymes. Although siderophores do not fit neatly into a definition of secondary metabolites, which are dispensable in laboratory growth conditions [29], these molecules are produced from clustered genes and are critical for pathogen growth in blood serum [30]. Because iron was included in the media used for the microarray study, we investigated whether the *Δlae* mutant was deficient in expression of siderophore gene cluster NRPSs under iron-limiting conditions.

As previously reported [29], low iron conditions induced transcriptional upregulation of several NRPS genes known or predicted to be involved in siderophore biosynthesis (Table 4). Normalized expression levels of the siderophore NRPSs in the low iron condition relative to high iron conditions were *NRPS2*/*sidC,* 2.245 ± 0.449; *NRPS3*/*sidE,* 68.595 ± 13.725; and *NRPS4*/*nps6*/*sidD,* 28.509 ± 4.704. *NRPS7* transcripts were not detectable in these experiments. In contrast to Reiber et al. [29], *NRPS3*/*sidE* showed the highest induction to the low iron conditions in our experiments. This discrepancy might be explained by differential sensitivity of the semiquantitative RT-PCR method used by Reiber et al. compared to our QRT-PCR methodology or subtle differences in culture conditions. We also noted that the complemented control strain with an ectopic copy of *laeA* showed increased expression of *NRPS3*/*sidE* in both low and high iron conditions.

**Table 4:**
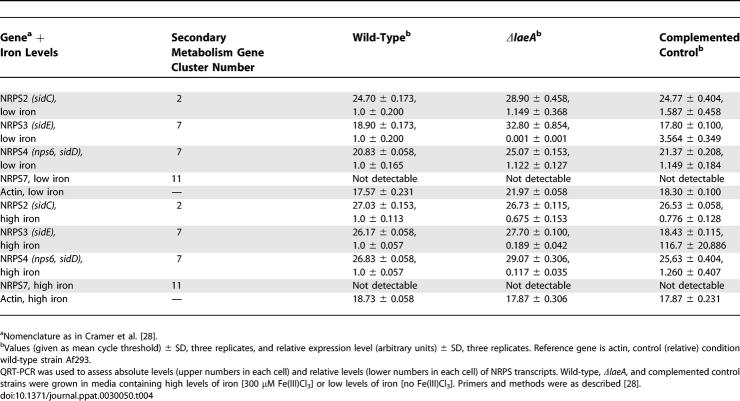
Real-Time RT-PCR Analysis of Select A. fumigatus Siderophore Biosynthesis NRPSs

Comparison of the *ΔlaeA* mutant and controls by QRT-PCR analysis indicated differential expression of *NRPS3*/*sidE* in low (inducing) iron conditions. In high iron conditions, *NRPS4*/*nps6*/*sidD, NRPS3/sidE,* and possibly *NRPS2*/*sidC* showed decreased expression in the *ΔlaeA* mutant (Table 4). Interestingly, *NRPS7* was not detectable in these experiments. In the low iron condition, expression of actin did decrease in the *ΔlaeA* mutant. However, the dramatic decrease in expression of *NRPS3*/*sidE* (1,000-fold less) seen in the *ΔlaeA* background strongly suggests that LaeA regulates the expression of at least this NRPS. Little is known about the function of SidE, although it has been speculated to be involved in siderophore biosynthesis on the basis of homology to SidC [29]. It remains to be determined whether *NRPS3*/*sidE* is involved in siderophore production, a process known to be critical to virulence [31,32], or whether it synthesizes an iron-responsive compound with a distinct function. Regardless of the function of SidE, these experiments show that LaeA is also involved in controlling expression of other secondary metabolite clusters not induced by the environmental conditions used in the microarray experiments.

### Secondary Metabolites under Control of LaeA

Cluster 18 (Figure 1) on Chromosome 6, strongly differentially expressed in *ΔlaeA*, encodes the genes required for gliotoxin biosynthesis. Gliotoxin is arguably the most well-studied mycotoxin produced by A. fumigatus. First identified in 1936, this compound has immunosuppressive properties in vitro [12] and in vivo [13,14], although its direct contribution to pathogenicity is only beginning to be understood [15–17]. Like all other compounds in the epipolythiodioxopiperazine class, gliotoxin is a cyclic dipeptide with an internal disulfide bridge that can undergo redox cycling (for a recent review, see [33]). Immunosuppressive activity of gliotoxin is due at least in part to negative regulation of the transcription factor nuclear factor–κB, which occurs by inhibition of proteasome-mediated degradation of the nuclear factor–κB inhibitor IκBα [34,35]. Gliotoxin is also known to be cytotoxic and can evoke both apoptotic [36–39] and necrotic [40,41] cell death. Recently, gliotoxin was shown to trigger the release of apoptogenic factors by the host mitochondrial protein Bak [42]. The secondary metabolism gene cluster responsible for gliotoxin production was recently identified by bioinformatic analysis [43] and has been experimentally confirmed [15,17]. Despite the known immunosuppressive activities of the molecule and its detection in blood serum of patients with invasive aspergillosis [44], three recent studies using genetic mutants of the gliotoxin gene cluster demonstrated that gliotoxin is not a virulence factor in murine models of invasive aspergillosis [15–17]. However, these same studies presented evidence that gliotoxin could adversely affect T cells, neutrophils, and mast cells and, we offer, likely acts synergistically with other LaeA-regulated toxins. The *ΔlaeA* mutant is impaired in gliotoxin production during growth in culture as well as growth in vivo in murine models of invasive aspergillosis [10,15], and the microarray results presented here confirm that LaeA strongly influences expression of genes in this cluster under the condition investigated.

Secondary metabolite cluster 1 on Chromosome 1, which is differentially expressed in *ΔlaeA,* contains an atypical NRPS called *Afpes1* that is required for virulence in an insect model of invasive aspergillosis [4]. *Afpes1* shows greatest homology to NRPSs that produce siderophores or destruxins, including one paralog required for virulence of the plant pathogen Alternaria brassicae [45]. However, the *Afpes1* cluster is thought to be unlikely to produce either of these compounds, because destruxin toxin has not been detected in A. fumigatus [4] and expression of *Afpes1* was not responsive to iron levels [4,21]. Deletion of *Afpes1* alters conidial morphology and hydrophobicity as well as melanin synthesis and results in increased susceptibility to reactive oxygen species, implying altered conidial melanin and/or rodlet composition [4]. Most of these characteristics are common to the *ΔlaeA* phenotype [11], possibly implicating a role of the *Afpes1* metabolite in the attenuated virulence of *ΔlaeA.*

A. fumigatus synthesizes several clavine ergot alkaloids, compounds that can be partial agonists or antagonists of serotonin, dopamine, and α-adrenalin receptors, thus affecting nervous, circulatory, reproductive, and immune system function [46]. The role of these compounds in invasive aspergillosis has not been determined. In addition to having the receptor-modulating activities mentioned, the festuclavine ergot alkaloid produced by A. fumigatus is cytostatic and is directly mutagenic in the Ames assay [47,48]. Recently, Coyle and Panaccione [25] showed that deletion of an A. fumigatus dimethylalleletryptophan synthase (DMAT synthase) homologous to *dmaW* of the ergot-producing species Claviceps purpurea eliminated all known ergot alkaloids, confirming its predicted function in the first committed step of ergot alkaloid production (i.e., addition of dimethylallyl diphosphate to l-tryptophan to result in 4-methylallyl-tryptophan). The biochemical activity of the A. fumgiatus DmaW enzyme was also confirmed by Unsöld and Li [22], who subsequently characterized a reverse prenyltransferase in the same gene cluster that converts fumigaclavine A to fumigaclavine C [23]. These genes are located in secondary metabolite gene cluster 4 on Chromosome 2, which is strongly differentially expressed in *ΔlaeA.*

Melanins found in conidia are one of the few described virulence factors in A. fumigatus [5,6,24]. Lack of melanins leads to increased susceptibility to reactive oxygen species produced by the host innate immune response during infection as well as altered (smooth) conidial morphology [5,7]. However, the scarcity of nonpigmented A. fumigatus spores in nature has drawn into question the clinical relevance of melanins as virulence factors [1]. Conidia of *ΔlaeA* are pigmented, but altered expression of *alb1* in the mutant has been reported previously and at least one unidentified spore metabolite is missing in *ΔlaeA* [11]. There is significant differential expression of the 1,8-dihydroxynapthalene–melanin gene cluster in *ΔlaeA* under the condition investigated in this study. Expression of this gene cluster is also regulated by cAMP/protein kinase A signaling [49] as is LaeA itself [10], perhaps a suggestion that in this case LaeA control of this cluster may be both directly and indirectly mediated by protein kinase A.

Additionally, a LaeA-regulated supercluster on Chromosome 8 is likely to produce multiple compounds. Recently, two genes in this cluster have been reported to encode biosynthetic enzymes for the tremorgenic mycotoxin fumitremorgin B and related compounds [26,27]. The cyclo-l-Trp-l-Pro derivative fumitremorgin B is cytotoxic, inhibiting cell cycle progression at G_2_/M, and thus has been of interest as a potential anticancer agent. The pathway involves generation of the cyclic dipeptide brevianamide F by the NRPS brevianamide synthetase [27], prenylation of brevianamide F by the prenyltransferase FtmPT1 to tryptrostatin B [26], and subsequent conversion in several steps to fumitremorgen B. Thus, LaeA-mediated influence on expression of *ftmPT1* and *ftmPT2* would govern the production of this entire class of diketopiperozine compounds. Once again, however, the specific effects of these compounds on pathogenicity during invasive aspergillosis are unknown.

The fact that LaeA promotes expression not only of these secondary metabolite gene clusters but an additional eight others confirms its role as a master controller of secondary metabolism. The importance of several of these compounds in toxicity studies also underscores relevance of LaeA during infection [11]. We suggest the possibility that virulence attributes are not influenced as much by individual metabolites as by the blend of LaeA-regulated toxins, which, in combination, may confer an advantage to the pathogen.

### LaeA Regulation of Evolutionarily Diverse Regions of the Genome

Comparative genomic analysis between A. fumigatus and related species indicates overlap between A. fumigatus–specific genes and genes differentially expressed in *ΔlaeA* (N. D. Fedorova and W. C. Nierman, unpublished data). In total, 68% of A. fumigatus secondary metabolite genes do not have orthologs in the closely related species A. clavatus (N. D. Fedorova and W. C. Nierman, unpublished data). Additional secondary metabolite genes do not have orthologs in more distantly related *Aspergilli* such as A. oryzae and A. nidulans [20]. The variability of secondary metabolite clusters may be explained by the fact that many of them are located in highly divergent telomere-proximal regions characterized by frequent chromosomal rearrangements [20,50]. For example, 54% of the clusters showing differential expression in *ΔlaeA* in the conditions described here were found within 300 kb of telomeres. It should be noted that, in addition to the secondary metabolite clusters, other genes with significantly lower expression in *ΔlaeA* also show some positional specificity within the genome but to a much lesser extent (unpublished data). Further analysis also showed that A. fumigatus telomere-proximal clusters tend to have larger numbers of genes than clusters located closer to the centromeres, suggesting that the former may accumulate additional genes more easily (N. D. Fedorova, J. R. Wortman, and W. C. Nierman, unpublished data).

Initial comparative genome analyses indicate that the telomere-proximal regions (and to a lesser extent, synteny breakpoints and intrasyntenic regions) appear to be a hotbed of diversity, not only between *Aspergillus* species but even between different strains of the same species [51,52]. The genomes of two A. fumigatus strains have been sequenced: the clinical isolate Af293 (by The Institute for Genomic Research, Rockville, Maryland, United States) and isolate CEA10 (under contract from Elitra Pharmaceutical by Celera Genomics and made available by Merck; B. Jiang and W. C. Nierman, personal communication). These strains show an overall divergence of 2%, and the majority of this variation is in telomere-proximal and synteny breakpoint regions. Similarly, microarray experiments also supported high divergence in these regions when Af293 was compared to the unsequenced A. fumigatus strains Af294 and Af71 [20].

Many secondary metabolite clusters appear to be associated with transposons (known to be active in A. fumigatus [51]) and transposase-like sequences (Table S3). The finding that these transposable elements often flank or are embedded in many of the clusters may represent one mechanism for generating the diversity of secondary metabolites in aspergilli. Whether or not there is a connection between LaeA function and transposon activity has yet to be established. In total, these analyses suggest that secondary metabolite clusters are located in the regions that undergo extensive rearrangements, which may result in subsequent alterations in secondary metabolite production and, therefore, have major impacts on niche adaptation between different species of fungi or between strains of the same species. Other examples include a non–aflatoxin-producing clade of *A. flavus,* better known as the food-fermenting A. oryzae used in the production of traditional Asian products such as miso and soy sauce, which may have arisen as a result of telomere-proximal rearrangements [53]. Similarly, genotypic variability between strains of Fusarium compactum also proved to be a major determinant of metabolite production and geographic distribution [54]. In *Fusarium graminearum,* the major cause of wheat and barley head blight, intraspecific polymorphic variations in a trichothecene mycotoxin gene cluster were correlated with chemotype differences, host range, and fitness [55].

In light of such examples, it is interesting to speculate about the role of LaeA in chemotype evolution and niche adaptation. It is possible that variation at any particular secondary metabolite gene cluster could result in less efficient control by LaeA. This potential has been demonstrated in A. nidulans [18]. Conversely, LaeA itself is a major target for comprehensive changes in the entire complement of secondary metabolites. The clustering of secondary metabolite biosynthetic genes has been suggested to reflect their evolutionary history [8,9,20,51,56,57]. Several models have been proposed to explain the establishment and maintenance of secondary metabolic gene clusters in filamentous fungi. The “selfish cluster” hypothesis proposes that selection occurs at the level of the cluster and promotes maintenance of the cluster as a unit, possibly through horizontal transfer events [56]. However, there is only limited evidence for widespread horizontal transfer of secondary metabolism gene clusters, with penicillin being a notable exception [58]. Alternative models suggest that clusters are maintained due to coregulation mechanisms, likely at the level of chromatin regulation [8,9]. LaeA may provide a mechanistic means of secondary metabolism gene cluster coregulation and maintenance. Certainly LaeA demonstrates a positional bias for local gene regulation, as transfer of genes into or out of a secondary metabolite cluster leads to respective gain or loss of transcriptional regulation by LaeA [18]. This has been speculated to occur through regulation of nucleosome positioning and heterochromatin formation [9].

### Conclusions

Our results confirm that LaeA plays a central role in regulation of chemical diversity in A. fumigatus. Furthermore, genomic regions that are transcriptionally controlled by LaeA are species and even strain specific, suggesting that they may serve as niche adaptation factors. The loss of *laeA* results in a great decrease in repertoire of secondary metabolites, which appears to impact the infection process. Therefore, LaeA constitutes a novel target for the production of an array of factors critical to success during pathogenesis. Furthermore, LaeA is a tool to identify metabolite gene clusters that may impact virulence, allowing the correlation of specific secondary metabolite clusters with virulence even in absence of knowledge about the mycotoxin itself.

## Materials and Methods

### Strains and growth conditions.

Three prototrophic A. fumigatus fungal strains were used in this study. Af293 (the wild-type clinical isolate used in the A. fumigatus genome sequencing project [20]), TJW54.2 *(ΔlaeA)* [11], and a complemented control strain TJW68.6 *(ΔlaeA* + *laeA)* [11] were grown in triplicate at 25 °C in liquid minimal media [59] with shaking (280 rpm) for 60 h. Profiles of secondary metabolites extracted from the media with chloroform were compared by thin-layer chromatography, and the results confirmed that the *ΔlaeA* strain showed reduced levels of multiple secondary metabolites under this condition ([10] and unpublished data). Total RNA was isolated from fungal mats, labeled, and hybridized with a DNA whole-genome amplicon microarray [20,60] in three independent biological replicates.

To analyze siderophore NRPS gene expression under low- or high-iron conditions, 50-ml liquid cultures were grown as described [29], with low-iron media containing 25 g/L glucose, 3.5 g/L (NH_4_)_2_SO_4_, 2.0 g/L KH_2_PO_4_, 0.5 g/L MgSO_4_ (heptahydrate), and 8 mg/L ZnSO_4_ (heptahydrate) (pH 6.3). High-iron media was identical except for the addition of Fe(III)Cl_3_ to a final concentration of 300 μM. Cultures were grown at 37 °C, 280 rpm, and samples were collected at 24 h postinoculation. All glassware was subjected to sequential treatment with 1 mM and 5% HCl as described [29].

### RNA isolation and microarray hybridization.

Total RNA was extracted from *Aspergillus* strains by use of TriZOL reagent (Invitrogen, http://www.invitrogen.com) according to the manufacturer's instructions. RNA was further purified by two extractions with phenol:chloroform:isoamyl alcohol (25:24:1) and then labeled with Cy-3 or Cy-5 dye and hybridized as previously described [20]. The generation of the whole genome array has been described [20].

### Relative expression levels of select NRPSs.

QRT-PCR was used to (1) confirm the expression level trends observed in the microarray experiment and (2) investigate NRPS gene expression under iron-limiting conditions. Expression of select NRPSs putatively regulated by LaeA was examined. Total RNA from two or three biological replicates was pooled in equal amounts (2 μg per sample) for each *Aspergillus* strain, wild-type AF293, TW54.2, and TW68.6, and treated with Ambion Turbo DNA-free DNase I (Ambion, http://www.ambion.com) to remove contaminating genomic DNA. A total of 500 ng of DNase I–treated total RNA from each sample was reverse transcribed with Superscript III reverse transcriptase (Invitrogen). Real-time RT-PCR was conducted with 20-μl reaction volumes with the iQ SYBR green supermix (Bio-Rad, http://www.bio-rad.com), 2 μl of a 1:6 dilution of first-strand cDNA, and 0.4 μl of each 10 μM primer stock. Primer sequences were previously reported [28]. No reverse transcriptase controls (NRT) were used to confirm elimination of contaminating genomic DNA. Real-time RT-PCR was performed using an iQ Cycler Real-Time PCR detection system (Bio-Rad). PCRs for each NRPS were done in triplicate and melt curve analysis was performed immediately following the PCR to confirm the absence of nonspecific amplification products and primer dimers. The relative expression levels of NRPS genes between A. fumigatus wild-type strain AF293, the *ΔlaeA* mutant, and the complemented control strain were calculated using 2^−ΔΔCt^ method with iQ cycler system software. All values were normalized to expression of the A. fumigatus actin gene and relative to the wild-type strain for each condition analyzed.

### Data analysis.

Gene expression ratios were determined for triplicate comparisons of (1) wild-type and *ΔlaeA* and (2) *ΔlaeA* and the complemented control strain. Prior to statistical analysis, the LOWESS normalization method was used to remove any systematic bias from the raw expression ratios [61]. Loci showing significantly different expression were identified using the SAM method for one-class designs that has been previously described in detail [19], implemented in the TM4 suite's MultiExperiment Viewer (http://www.tm4.org) [62,63]. This allowed identification of genes whose mean expression across experiments is significantly different from a user-specified mean (log_2_ = 0, corresponding to identical mRNA levels in the mutant and wild-type strains). Genes with scores above the significance threshold and exceeding the cutoff value of zero for the false discovery rate (the most conservative setting) were designated as significantly differentially expressed between mutant and wild-type. The delta value cutoff in SAM was chosen to capture the maximum number of significant genes while maintaining the reported estimated false discovery rate at zero. Genes down-regulated in *ΔlaeA* were further analyzed by the Expression Analysis Systematic Explorer (EASE) [64] within TM4 to identify overrepresented Gene Ontology terms and Pfam domains. Fisher's exact test probabilities and step-down Bonferroni corrected probabilities are reported from the EASE analysis to indicate which terms are overrepresented in the down-regulated gene set.

## Supporting Information

Figure S1 Expression Ratio Heat Map A subset of LaeA-regulated genes (total of 409), including 174 genes up-regulated in *ΔlaeA* and 235 genes down-regulated in *ΔlaeA*. First three columns show raw expression ratio values of *ΔlaeA* versus wild-type in three independent biological replicates. Last three columns show expression ratios of wild-type versus a complemented *ΔlaeA* strain. Color-coded expression ratio scale is shown at top.
(1.9 MB DOC)

Figure S2 Functional Categories of Genes Showing Significantly Different Gene Expression in *ΔlaeA* versus Wild-Type, as Percentages of Total Categories are as indicated and described in detail in Table 1.
(1.9 MB DOC)

Table S1 Statistical Analysis of Overrepresented Gene Ontology Categories among Genes Down-regulated in *ΔlaeA*
Numbers of genes in the indicated Gene Ontology categories were subjected to statistical analysis by EASE [64] to identify categories overrepresented compared with the whole genome data set. Fisher's exact test probabilities and Bonferroni corrected (step-down) probabilities (Bonferroni SD Corr.) are reported from the EASE analysis. Only categories with Fisher's exact test probabilities below 5.00E-02 were included.
(41 KB DOC)

Table S2 Statistical Analysis of Overrepresented Pfam Protein Domains among Genes Significantly Down-regulated in *ΔlaeA*
Numbers of genes in the indicated Pfam protein domain categories [65] were subjected to statistical analysis by EASE [64] to identify categories overrepresented compared with the whole genome data set. Fisher's exact test probabilities and Bonferroni corrected (step-down) probabilities (Bonferroni SD Corr.) are reported from the EASE analysis. Only categories with Fisher's exact test probabilities below 5.00E-02 were included.
(35 KB DOC)

Table S3 Expression Ratio Values for All Secondary Metabolite Gene Clusters Normalized expression ratio values are shown for *ΔlaeA* versus wild-type (column labeled “*ΔlaeA*”) or complemented control strain versus wild-type (column labeled “comp”). Expression ratios are shown for three independent biological replicates, followed by average values (last two columns). Bold text in “Gene Name” category indicates critical genes in the cluster such as polyketide synthases or NRPSs.
(846 KB DOC)

## Abbreviations

NRPS: nonribosomal peptide synthetase
QRT-PCR: quantitative reverse-transcription PCR
RT: reverse-transcription
SAM: significance analysis of microarrays
