# Supplementary material for: Transcriptional Regulation of Chemical Diversity in Aspergillus fumigatus by LaeA
Source: PLoS Pathog. 2007 Apr 13;3(4):e50. doi: 10.1371/journal.ppat.0030050 (PMC1851976; doi:10.1371/journal.ppat.0030050)
Supplement: Supplementary file 1 [file ppat.0030050.sg001.doc]

**Supplemental Figure 1. Expression ratio heat map.** A subset of LaeA-regulated genes (409 total), including 174 genes up-regulated in *ΔlaeA* and 235 genes down-regulated in *ΔlaeA*. First three columns, raw expression ratio values of *ΔlaeA* vs wild-type in three independent biological replicates. Last three columns, expression ratios of wild-type versus a complemented *ΔlaeA* strain. Color-coded expression ratio scale is shown at top.
