# Supplementary material for: Transcriptional Regulation of Chemical Diversity in Aspergillus fumigatus by LaeA
Source: PLoS Pathog. 2007 Apr 13;3(4):e50. doi: 10.1371/journal.ppat.0030050 (PMC1851976; doi:10.1371/journal.ppat.0030050)
Supplement: Supplementary file 2 [file ppat.0030050.sg002.doc]

**Supplemental Figure 2. Functional categories of genes showing significantly different gene expression in *ΔlaeA* versus wild-type, as percentages of total.** Categories are as indicated and described in detail in Table 1.
