# Supplementary material for: Transcriptional Regulation of Chemical Diversity in Aspergillus fumigatus by LaeA
Source: PLoS Pathog. 2007 Apr 13;3(4):e50. doi: 10.1371/journal.ppat.0030050 (PMC1851976; doi:10.1371/journal.ppat.0030050)
Supplement: Supplementary file 3 [file ppat.0030050.st001.doc]

**Supplemental Table 1. Statistical analysis of over-represented gene ontology categories amongst genes down-regulated in *ΔlaeA*.** Numbers of genes in the indicated gene ontology (GO) categories were subjected to statistical analysis by the Expression Analysis Systematic Explorer (EASE) [64] to identify categories over-represented as compared to the whole genome data set. Fisher’s exact test probabilities and Bonferroni corrected (Step Down) probabilities (Bonferroni SD Corr.) are reported from the EASE analysis. Only categories with Fisher’s exact test probabilities below 5.00E-02 were included.

| **Gene Ontology (GO) term** | **GO type** | **Fisher's Exact** | **Bonferroni Step Down Correction** |
| --- | --- | --- | --- |
| secondary metabolism | Process | 3.37E-08 | 2.62E-05 |
| sulfur metabolism | Process | 1.77E-06 | 1.37E-03 |
| nitrogen compound metabolism | Process | 2.47E-06 | 1.91E-03 |
| pathogenesis | Process | 2.54E-06 | 1.97E-03 |
| amino acid metabolism | Process | 9.91E-06 | 7.64E-03 |
| nonribosomal peptide biosynthesis | Process | 1.05E-05 | 8.07E-03 |
| choline transport | Process | 1.76E-05 | 1.35E-02 |
| aromatic compound metabolism | Process | 6.33E-05 | 4.79E-02 |
| amine metabolism | Process | 9.09E-05 | 6.88E-02 |
| heterocycle metabolism | Process | 3.73E-03 | 1.00E+00 |
| response to chemical stimulus | Process | 5.38E-03 | 1.00E+00 |
| cell wall organization and biogenesis | Process | 6.84E-03 | 1.00E+00 |
| carboxylic acid metabolism | Process | 7.92E-03 | 1.00E+00 |
| oxygen and reactive oxygen species metabolism | Process | 9.52E-03 | 1.00E+00 |
| amine transport | Process | 1.07E-02 | 1.00E+00 |
| sterol biosynthesis | Process | 1.20E-02 | 1.00E+00 |
| plasma membrane | Component | 1.13E-05 | 8.69E-03 |
| cell wall (sensu Fungi) | Component | 4.83E-03 | 1.00E+00 |
