# Supplementary material for: Transcriptional Regulation of Chemical Diversity in Aspergillus fumigatus by LaeA
Source: PLoS Pathog. 2007 Apr 13;3(4):e50. doi: 10.1371/journal.ppat.0030050 (PMC1851976; doi:10.1371/journal.ppat.0030050)
Supplement: Supplementary file 4 [file ppat.0030050.st002.doc]

**Supplemental Table 2. Statistical analysis of over-represented PFAM protein domains amongst genes significantly down-regulated in *ΔlaeA.*** Numbers of genes in the indicated PFAM protein domain categories [65] were subjected to statistical analysis by the Expression Analysis Systematic Explorer (EASE) [64] to identify categories over-represented as compared to the whole genome data set. Fisher’s exact test probabilities and Bonferroni corrected (Step-Down) probabilities (Bonferroni SD Corr.) are reported from the EASE analysis. Only categories with Fisher’s exact test probabilities below 5.00E-02 were included.

| **PFAM Domain** | **Putative Function** | **Fisher's Exact** | **Bonferroni Step Down Correction** |
| --- | --- | --- | --- |
| Cytochrome P450 | metabolism | 7.41E-11 | 1.46E-08 |
| AMP-binding enzyme | AMP binding | 1.97E-05 | 3.82E-03 |
| FAD binding domain | FAD binding | 1.46E-03 | 2.76E-01 |
| Phosphopantetheine attachment site | secondary metabolism | 6.85E-08 | 1.34E-05 |
| Condensation domain | secondary metabolism | 3.38E-07 | 6.60E-05 |
| Amino acid permease | transport | 9.29E-04 | 1.78E-01 |
| O-methyltransferase | secondary metabolism | 8.52E-04 | 1.65E-01 |
| Glutathione S-transferase, C-terminal domain | secondary metabolism | 8.52E-04 | 1.65E-01 |
| Acyl transferase domain | secondary metabolism | 2.59E-03 | 4.87E-01 |
| Beta-ketoacyl synthase | secondary metabolism | 4.58E-03 | 8.57E-01 |
| Pyridine nucleotide-disulphide oxidoreductase | nucleotide metabolism | 5.89E-03 | 1.00E+00 |
