# Supplementary material for: Transcriptional Regulation of Chemical Diversity in Aspergillus fumigatus by LaeA
Source: PLoS Pathog. 2007 Apr 13;3(4):e50. doi: 10.1371/journal.ppat.0030050 (PMC1851976; doi:10.1371/journal.ppat.0030050)
Supplement: Supplementary file 5 [file ppat.0030050.st003.doc]

**Supplemental Table 3. Expression ratio values for all secondary metabolite gene clusters.** Normalized expression ratio values are shown for *ΔlaeA* vs wild-type (column labeled “*ΔlaeA*”) or complemented control strain versus wild-type (column labeled “comp”). Expression ratios are shown for three independent biological replicates, followed by average values (last two columns). Bold text in “Gene Name” category indicates critical genes in the cluster such as polyketide synthases or nonribosomal peptide synthetases.

| **Cluster** | **Locus ID** | **Gene Name [function]** | **ΔlaeA1** | **ΔlaeA2** | **ΔlaeA3** | **comp1** | **comp2** | **comp3** | **ΔlaeA** | **comp** |
| --- | --- | --- | --- | --- | --- | --- | --- | --- | --- | --- |
|  | **Afu1g10350** | phosphoglycerate kinase PgkA, putative | 0.29 | 0.91 | 0.60 | -0.30 | -0.02 | -0.28 | 0.60 | -0.20 |
| 1 | Afu1g10360 | conserved hypothetical protein | -0.01 | 0.06 | 0.08 | 0.08 | 0.02 | 0.15 | 0.05 | 0.08 |
| 1 | **Afu1g10370** | MSF drug transporter, putative | 0.24 | 0.29 | 0.25 | 0.43 | 0.05 | 0.38 | 0.26 | 0.29 |
| 1 | **Afu1g10380** | **nonribosomal peptide synthase Afpes1** | 2.40 | 1.92 | 2.36 | 0.07 | -0.27 | 0.14 | 2.23 | -0.02 |
| 1 | **Afu1g10390** | ABC multidrug transporter, putative | 2.39 | 1.06 | 1.78 | -0.01 | -0.35 | 0.13 | 1.74 | -0.08 |
|  |  |  |  |  |  |  |  |  |  |  |
| 2 | Afu1g17640 | C6 transcription factor RegA |  |  |  |  |  |  |  |  |
| 2 | Afu1g17650 | short chain dehydrogenase, putative | #DIV/0! | #DIV/0! | -0.12 | #DIV/0! | 0.23 | #DIV/0! | -0.12 | 0.23 |
| 2 | Afu1g17660 | histidinol dehydrogenase, putative |  |  |  |  |  |  |  |  |
| 2 | Afu1g17670 | salicylate hydroxylase, putative |  |  |  |  |  |  |  |  |
| 2 | Afu1g17680 | MFS transporter, putative | #DIV/0! | -1.20 | -1.03 | #DIV/0! | #DIV/0! | #DIV/0! | -1.12 |  |
| 2 | **Afu1g17690** | FAD binding oxidoreductase, putative | 0.57 | 0.44 | 0.61 | 0.12 | 0.04 | 0.20 | 0.54 | 0.12 |
| 2 | Afu1g17700 | short chain dehydrogenase, putative | |  |  |  |  |  |  |  |
| 2 | **Afu1g17705** | Afut1-like retrotransposon |  |  |  |  |  |  |  |  |
| 2 | Afu1g17710 | C6 transcription factor, putative | -0.12 | #DIV/0! | #DIV/0! | 0.13 | -0.31 | #DIV/0! | -0.12 | -0.09 |
| 2 | Afu1g17720 | oxidoreductase, putative |  |  |  |  |  |  |  |  |
| 2 | Afu1g17725 | cytochrome P450 oxidoreductase, putative | |  |  |  |  |  |  |  |
| 2 | Afu1g17730 | zinc binding enoyl reductase, putative | #DIV/0! | #DIV/0! | #DIV/0! | 0.47 | 0.09 | 0.13 |  | 0.23 |
| 2 | Afu1g17740 | **polyketide synthase, putative** |  |  |  |  |  |  |  |  |
|  |  |  |  |  |  |  |  |  |  |  |
|  |  |  |  |  |  |  |  |  |  |  |
|  |  | **conidial pigment biosynthesis cluster** | |  |  |  |  |  |  |  |
| 3 | **Afu2g17510** | conserved hypothetical protein |  |  |  |  |  |  | 0.23 | -0.26 |
| 3 | Afu2g17520 | GNAT family N-acetyltransferase, putative | 0.19 | #DIV/0! | #DIV/0! | #DIV/0! | 0.03 | #DIV/0! | 0.19 | 0.03 |
| 3 | **Afu2g17530** | **conidial pigment biosynthesis oxidase Arb2/brown2** | 2.55 | 1.95 | 1.74 | 0.54 | 0.64 | 0.46 | 2.08 | 0.54 |
| 3 | **Afu2g17540** | **pigment biosynthesis oxidase Abr1/brown 1** | 3.69 | 3.38 | 2.82 | 0.55 | 1.10 | 0.48 | 3.30 | 0.71 |
| 3 | **Afu2g17550** | **pigment biosynthesis protein Ayg1** | 3.65 | 3.20 | 3.40 | 0.75 | 0.80 | 0.91 | 3.42 | 0.82 |
| 3 | **Afu2g17560** | **1,3,6,8-tetrahydroxynaphthalene reductase** | 4.51 | 4.30 | 4.32 | 1.06 | 1.00 | 1.04 | 4.38 | 1.03 |
| 3 | **Afu2g17570** | hypothetical protein |  |  |  |  |  |  | 1.53 | 0.52 |
| 3 | **Afu2g17580** | conidial pigment biosynthesis scytalone dehydratase Arp1 | 4.62 | 4.27 | 3.92 | 0.58 | 1.03 | 0.66 | 4.27 | 0.76 |
| 3 | **Afu2g17590** | conserved hypothetical protein |  |  |  |  |  |  | 0.42 | 0.44 |
| 3 | **Afu2g17600** | **polyketide synthetase PksP** | 4.13 | 3.59 | 3.99 | 1.26 | 0.73 | 1.26 | 3.90 | 1.08 |
|  |  |  |  |  |  |  |  |  |  |  |
| 4 | **Afu2g17960** | NADPH dehydrogenase, putative | 1.00 | #DIV/0! | 0.88 | 0.39 | 0.34 | 0.38 | 0.94 | 0.37 |
| 4 | **Afu2g17970** | conserved hypothetical protein | 1.90 | 1.80 | 1.35 | 0.63 | 0.80 | 0.71 | 1.68 | 0.71 |
| 4 | Afu2g17980 | cytochrome P450 demethylase, putative | |  |  |  |  |  |  | 0.18 |
| 4 | Afu2g17990 | **dimethylallyl tryptophan synthase, putative** | #DIV/0! | #DIV/0! | #DIV/0! | 0.18 | #DIV/0! | #DIV/0! |  |  |
| 4 | **Afu2g18000** | short chain dehydrogenase, putative | 2.63 | 2.79 | 2.46 | 0.82 | 1.13 | 0.78 | 2.63 | 0.91 |
| 4 | **Afu2g18010** | cytochrome P450 monooxygenase, putative | 1.84 | #DIV/0! | 1.44 | 0.64 | 0.51 | 0.76 | 1.64 | 0.63 |
| 4 | Afu2g18020 | transferase family protein |  |  |  |  |  |  |  |  |
| 4 | **Afu2g18030** | catalase, putative | 3.92 | 3.29 | 3.26 | 0.79 | 0.70 | 0.79 | 3.49 | 0.76 |
| 4 | **Afu2g18040** | **dimethylallyl tryptophan synthase, putative** | 2.84 | 2.78 | 2.64 | 0.66 | 1.18 | 0.67 | 2.75 | 0.84 |
| 4 | **Afu2g18050** | FAD binding oxidoreductase, putative | 0.80 | 0.25 | 0.22 | 0.29 | 0.31 | -0.05 | 0.42 | 0.19 |
| 4 | **Afu2g18060** | conserved hypothetical protein | 2.01 | 1.66 | 1.61 | 0.56 | 0.69 | 0.56 | 1.76 | 0.60 |
| 4 | Afu2g18070 | metalloproteinase, putative |  |  |  |  |  |  |  |  |
|  | **Afu2g18080** | LINE-1 class reverse transcriptase, RNaseH, putative | | |  |  |  |  | 0.24 | 0.27 |
|  |  |  |  |  |  |  |  |  |  |  |
| 5 | **Afu3g01290** | AMID-like mitochondrial oxidoreductase, putative | 1.60 | 0.48 | 1.30 | 0.74 | 0.59 | 0.51 | 1.12 | 0.61 |
| 5 | Afu3g01300 | conserved hypothetical protein | -0.65 | -0.68 | #DIV/0! | #DIV/0! | 0.14 | #DIV/0! | -0.66 |  |
| 5 | Afu3g01310 | hypothetical protein |  |  |  |  |  |  |  |  |
| 5 | Afu3g01320 | homocysteine S-methyltransferase, putative | -0.06 | -0.35 | -0.06 | 0.14 | -0.20 | 0.17 | -0.16 | 0.04 |
| 5 | Afu3g01330 | class II aldolase/adducin domain protein | -0.87 | -1.22 | -0.83 | 0.36 | -0.03 | 0.39 | -0.97 | 0.24 |
| 5 | Afu3g01340 | DUF636 domain protein | -0.32 | -0.28 | -0.13 | 0.05 | 0.19 | -0.09 | -0.24 | 0.05 |
| 5 | Afu3g01350 | proteinase, putative | 0.08 | -0.22 | -0.10 | -0.06 | -0.11 | 0.16 | -0.08 | -0.01 |
| 5 | Afu3g01360 | siderochrome-iron transporter, putative | -0.40 | #DIV/0! | #DIV/0! | 0.18 | #DIV/0! | #DIV/0! | -0.40 | 0.18 |
| 5 | Afu3g01370 | MFS transporter, putative | -1.08 | -0.72 | -0.98 | 0.28 | -0.26 | -0.11 | -0.93 | -0.03 |
| 5 | Afu3g01380 | hypothetical protein |  |  |  |  |  |  |  |  |
| 5 | **Afu3g01390** | hypothetical protein |  |  |  |  |  |  | 0.48 | 0.16 |
| 5 | **Afu3g01400** | ABC multidrug transporter, putative | 2.52 | 2.20 | 2.25 | 0.64 | 0.36 | 0.46 | 2.32 | 0.49 |
| 5 | **Afu3g01410** | **polyketide synthase, putative** | 2.43 | 0.93 | 2.17 | 0.86 | -0.52 | 0.96 | 1.84 | 0.44 |
| 5 | **Afu3g01420** | DUF341 domain oxidoreductase, putative | 0.95 | 0.60 | 0.95 | 0.33 | 0.26 | 0.35 | 0.83 | 0.31 |
| 5 | Afu3g01430 | benzodiazepine receptor family protein | -0.27 | -0.31 | 0.13 | 0.18 | -0.37 | 0.44 | -0.15 | 0.08 |
| 5 | **Afu3g01440** | DUF1212 domain membrane protein | 0.94 | 0.35 | 0.87 | 0.22 | -0.24 | 0.31 | 0.72 | 0.10 |
| 5 | Afu3g01450 | 3-methyl-2-oxobutanoate dehydrogenase, putative | -1.46 | -1.59 | -1.48 | 0.17 | 0.32 | 0.09 | -1.51 | 0.19 |
| 5 | Afu3g01460 | salicylate hydroxylase, putative | -0.31 | -0.33 | #DIV/0! | -0.31 | #DIV/0! | 0.01 | -0.32 | -0.15 |
| 5 | Afu3g01470 | conserved hypothetical protein | -1.12 | -1.10 | -1.05 | -0.07 | 0.23 | -0.11 | -1.09 | 0.01 |
| 5 | Afu3g01480 | conserved hypothetical protein | -1.28 | -1.16 | -1.17 | -0.17 | 0.23 | -0.12 | -1.20 | -0.02 |
| 5 | Afu3g01490 | alcohol dehydrogenase, putative | -1.88 | -1.93 | -1.78 | -0.13 | -0.17 | -0.26 | -1.87 | -0.19 |
| 5 | Afu3g01500 | integral membrane protein | -1.76 | -2.73 | -1.68 | 0.45 | -0.36 | 0.44 | -2.06 | 0.18 |
| 5 | Afu3g01510 | C6 transcription factor, putative | -0.01 | -0.44 | -0.20 | 0.01 | -0.18 | -0.15 | -0.21 | -0.11 |
| 5 | **Afu3g01520** | MFS toxin efflux pump, putative | 0.37 | 0.26 | 0.07 | 0.12 | 0.05 | 0.01 | 0.23 | 0.06 |
| 5 | Afu3g01530 | phosphatidylglycerol specific phospholipase, putative | -0.56 | -0.76 | -0.56 | 0.20 | -0.07 | -0.07 | -0.63 | 0.02 |
| 5 | Afu3g01540 | MFS monocarboxylate transporter, putative | -0.31 | -0.40 | -0.19 | -0.21 | -0.31 | 0.05 | -0.30 | -0.16 |
| 5 | Afu3g01560 | amino acid permease, putative |  |  |  |  |  |  |  |  |
| 5 | Afu3g01580 | GMC oxidoreductase, putative | -1.30 | -0.64 | -1.24 | 0.48 | 0.53 | 0.36 | -1.06 | 0.46 |
| 5 | Afu3g01590 | conserved hypothetical protein | -0.45 | -0.24 | -0.30 | 0.56 | 0.19 | 0.53 | -0.33 | 0.43 |
| 5 | **Afu3g01600** | monooxygenase, putative | 1.68 | 1.27 | 1.33 | 0.09 | 0.18 | -0.12 | 1.43 | 0.05 |
|  |  |  |  |  |  |  |  |  |  |  |
| 6 | **Afu3g02520** | MSF multidrug transporter, putative | 0.80 | 0.74 | 0.55 | 0.17 | 0.55 | 0.10 | 0.70 | 0.27 |
| 6 | Afu3g02530 | **polyketide synthase, putative** | 0.86 | #DIV/0! | 0.61 | 0.15 | 0.49 | 0.23 | 0.74 | 0.29 |
| 6 | Afu3g02540 | hypothetical protein | -0.90 | -0.58 | -0.27 | 0.16 | 0.00 | 0.37 | -0.58 | 0.18 |
| 6 | **Afu3g02550** | conserved hypothetical protein | 0.15 | 0.41 | 0.31 | 0.65 | 0.91 | 0.63 | 0.29 | 0.73 |
| 6 | Afu3g02560 | hypothetical protein |  |  |  |  |  |  |  |  |
| 6 | Afu3g02570 | **polyketide synthase, putative** | 0.10 | 0.25 | 0.11 | 0.29 | 0.11 | 0.40 | 0.15 | 0.27 |
| 6 | Afu3g02580 | short-chain dehydrogenase/reductase family protein, putative | -0.01 | -0.21 | 0.05 | 0.00 | 0.12 | -0.04 | -0.06 | 0.03 |
| 6 | **Afu3g02590** | C6 transcription factor, putative | 0.63 | 0.01 | 0.05 | -0.03 | -0.02 | -0.21 | 0.23 | -0.09 |
| 6 | Afu3g02600 | conserved hypothetical protein | -0.30 | 0.03 | -0.40 | -0.28 | -0.23 | -0.32 | -0.23 | -0.28 |
| 6 | Afu3g02610 | MFS transporter, putative |  |  |  |  |  |  |  |  |
| 6 | Afu3g02620 | phenol hydroxylase, putative | 0.07 | 0.16 | 0.25 | -0.10 | -0.15 | 0.09 | 0.16 | -0.05 |
| 6 | Afu3g02630 | conserved hypothetical protein | -0.36 | -0.23 | -0.35 | -0.28 | 0.30 | -0.37 | -0.31 | -0.11 |
| 6 | **Afu3g02640** | nucleoside-diphosphate-sugar epimerase family protein | 0.51 | 0.30 | 0.46 | -0.27 | -0.17 | -0.04 | 0.42 | -0.16 |
| 6 | Afu3g02650 | MYB DNA binding domain protein | -0.61 | -0.39 | -0.37 | 0.06 | 0.22 | 0.07 | -0.46 | 0.12 |
| 6 | Afu3g02660 | hypothetical protein |  |  |  |  |  |  |  |  |
| 6 | Afu3g02670 | **nonribosomal peptide synthetase, putative** | -0.11 | 0.14 | -0.01 | -0.14 | 0.09 | -0.08 | 0.01 | -0.05 |
| 6 | Afu3g02680 | conserved hypothetical protein | -0.06 | 0.13 | -0.06 | -0.16 | -0.19 | -0.11 | 0.00 | -0.16 |
| 6 | Afu3g02690 | conserved hypothetical protein |  |  |  |  |  |  |  |  |
| 6 | Afu3g02700 | transcription factor, putative | #DIV/0! | #DIV/0! | #DIV/0! | -0.08 | #DIV/0! | #DIV/0! |  | -0.08 |
| 6 | Afu3g02710 | zinc-binding oxidoreductase, putative | 0.05 | 0.31 | -0.09 | -0.04 | 0.29 | -0.10 | 0.09 | 0.05 |
| 6 | Afu3g02720 | MFS transporter, putative | -0.12 | -0.07 | -0.19 | -0.13 | 0.03 | -0.01 | -0.13 | -0.04 |
|  |  |  |  |  |  |  |  |  |  |  |
| 7 | Afu3g03090 | Taf1 transposase, putative |  |  |  |  |  |  |  |  |
| 7 | **Afu3g03190** | MFS transporter, putative | 0.34 | 0.21 | 0.55 | 0.14 | 0.02 | -0.18 | 0.37 | -0.01 |
| 7 | Afu3g03200 | cysteine hydrolase family protein |  |  |  |  |  |  |  |  |
| 7 | Afu3g03210 | hypothetical protein | -0.24 | 0.11 | -0.16 | 0.04 | 0.37 | -0.24 | -0.10 | 0.06 |
| 7 | Afu3g03220 | conserved hypothetical protein | -0.51 | #DIV/0! | #DIV/0! | -0.14 | -0.07 | #DIV/0! | -0.51 | -0.11 |
| 7 | Afu3g03230 | hypothetical protein | -0.91 | -1.09 | -0.74 | 0.11 | -0.15 | -0.04 | -0.91 | -0.03 |
| 7 | Afu3g03240 | MFS monocarboxylate transporter, putative | 0.00 | #DIV/0! | -0.33 | -0.27 | -0.02 | #DIV/0! | -0.16 | -0.15 |
| 7 | Afu3g03250 | aldehyde dehydrogenase family protein, putative | -0.19 | -0.15 | -0.31 | -0.13 | #DIV/0! | #DIV/0! | -0.21 |  |
| 7 | Afu3g03260 | hypothetical protein |  |  |  |  |  |  | -2.14 | -1.88 |
| 7 | Afu3g03270 | isochorismatase family hydrolase, putative | #DIV/0! | #DIV/0! | #DIV/0! | 0.05 | -0.26 | #DIV/0! |  | -0.10 |
| 7 | **Afu3g03280** | FAD binding monooxygenase, putative | 1.69 | 1.08 | 1.88 | 0.59 | -0.29 | 0.80 | 1.55 | 0.37 |
| 7 | Afu3g03290 | hypothetical protein | -2.08 | -1.75 | -1.66 | -0.48 | -0.22 | -0.38 | -1.83 | -0.36 |
| 7 | Afu3g03300 | FAD binding domain protein | -0.44 | -0.21 | -0.21 | -0.61 | -0.47 | -0.27 | -0.29 | -0.45 |
| 7 | Afu3g03310 | RTA1 domain protein | 0.21 | -0.13 | -0.22 | -0.07 | 0.16 | -0.33 | -0.05 | -0.08 |
| 7 | Afu3g03315 | C6 transcription factor, putative |  |  |  |  |  |  |  |  |
| 7 | Afu3g03320 | MFS monocarboxylate transporter, putative | #DIV/0! | -0.15 | #DIV/0! | -0.02 | #DIV/0! | #DIV/0! | -0.15 | -0.02 |
| 7 | Afu3g03330 | mitochondrial enoyl reductase, putative | -0.21 | 0.06 | -0.18 | -0.21 | -0.46 | -0.13 | -0.11 | -0.27 |
| 7 | **Afu3g03340** | hypothetical protein |  |  |  |  |  |  | 0.52 | -0.11 |
| 7 | **Afu3g03350** | **nonribosomal peptide synthase SidE** | 0.65 | 0.30 | 0.38 | 0.00 | -0.14 | 0.09 | 0.44 | -0.02 |
| 7 | **Afu3g03360** | hypothetical protein |  |  |  |  |  |  | 0.43 | -0.02 |
| 7 | Afu3g03370 | cell surface protein, putative | 0.15 | 0.30 | -0.12 | 0.17 | 0.56 | 0.03 | 0.11 | 0.25 |
|  | Afu3g03380 | maltose O-acetyltransferase, putative | 0.43 | -0.22 | #DIV/0! | -0.04 | #DIV/0! | #DIV/0! | 0.11 | -0.04 |
|  |  |  |  |  |  |  |  |  |  |  |
|  |  |  |  |  |  |  |  |  |  |  |
|  |  | **ETP toxin cluster** |  |  |  |  |  |  |  |  |
| 8 | Afu3g12870 | GTPase activating protein (Gyp3), putative | 0.05 | 0.23 | -0.09 | -0.03 | 0.03 | -0.28 | 0.06 | -0.10 |
| 8 | Afu3g12880 | hypothetical protein |  |  |  |  |  |  | 3.21 | 0.52 |
| 8 | **Afu3g12890** | C6 transcription factor (GliZ), putative | 3.37 | 2.98 | 3.30 | 0.50 | 0.42 | 0.66 | 5.03 | 1.45 |
| 8 | **Afu3g12900** | MFS monocarboxylate transporter (Mch4), putative | 5.15 | 5.05 | 4.89 | 1.67 | 1.25 | 1.43 |  |  |
| 8 | **Afu3g12910** | O-methyltransferase (GliM), putative | 3.92 | 3.66 | 3.40 | 0.70 | 0.47 | 0.74 | 3.66 | 0.64 |
| 8 | **Afu3g12920** | **nonribosomal peptide synthase (GliP), putative** | 2.79 | 2.78 | 2.55 | 0.69 | 0.67 | 0.61 | 2.71 | 0.66 |
| 8 | **Afu3g12930** | **dimethylallyl tryptophan synthase (GliD), putative** | 2.72 | 2.89 | 2.29 | 0.36 | 0.90 | 0.37 | 2.63 | 0.55 |
| 8 | **Afu3g12940** | C6 transcription factor, putative | 0.25 | -0.03 | 0.56 | 0.12 | -0.12 | 0.26 | 0.26 | 0.09 |
| 8 | **Afu3g12950** | FAD binding domain protein | 3.34 | 3.32 | 3.02 | 0.53 | 0.85 | 0.42 | 3.23 | 0.60 |
| 8 | **Afu3g12960** | cytochrome P450 monooxigenase (GliC), putative | 4.42 | 3.76 | 4.03 | 0.66 | 1.09 | 0.64 | 4.07 | 0.80 |
| 8 | Afu3g12970 | DUF580 domain protein | -0.07 | -0.62 | -0.11 | 0.50 | 0.11 | 0.62 | -0.27 | 0.41 |
| 8 | **Afu3g12980** | extracellular serine rich protein | 0.81 | 0.54 | #DIV/0! | 0.67 | 0.33 | 0.21 | 0.67 | 0.40 |
| 8 | Afu3g12990 | conserved proline-rich protein | -0.18 | 0.07 | -0.27 | 0.04 | 0.19 | -0.02 | -0.12 | 0.07 |
| 8 | Afu3g13000 | BCS1-like ATPase, putative | 0.07 | 0.55 | -0.09 | -0.17 | 0.42 | -0.27 | 0.18 | -0.01 |
| 8 | **Afu3g13010** | conserved hypothetical protein | 1.14 | 1.11 | 1.25 | 0.29 | 0.45 | 0.29 | 1.17 | 0.34 |
|  |  |  |  |  |  |  |  |  |  |  |
| 9 | **Afu3g13580** | dynamin GTPase, putative | 2.49 | 1.62 | 2.24 | 0.86 | 1.16 | 0.85 | 2.12 | 0.96 |
| 9 | Afu3g13590 | conserved hypothetical protein | -0.05 | 0.29 | 0.15 | -0.01 | 0.02 | 0.08 | 0.13 | 0.03 |
| 9 | Afu3g13600 | C6 transcription factor, putative | 0.00 | 0.16 | 0.23 | 0.03 | #DIV/0! | #DIV/0! | 0.13 | 0.03 |
| 9 | Afu3g13610 | oxidoreductase, zinc-binding, putative | 0.09 | -0.38 | 0.58 | 0.27 | 0.20 | 0.22 | 0.10 | 0.23 |
| 9 | **Afu3g13620** | conserved hypothetical protein | 1.08 | 0.49 | 0.84 | 0.77 | 0.30 | 0.64 | 0.80 | 0.57 |
| 9 | Afu3g13630 | conserved hypothetical protein |  |  |  |  |  |  |  |  |
| 9 | Afu3g13640 | extracellular serine-rich protein | -0.17 | #DIV/0! | #DIV/0! | -0.11 | 0.03 | #DIV/0! | -0.17 | -0.04 |
| 9 | Afu3g13650 | integral membrane protein | #DIV/0! | 0.27 | -0.08 | 0.07 | 0.01 | -0.08 | 0.10 | 0.00 |
| 9 | Afu3g13660 | Ctr copper transporter family protein | 0.04 | #DIV/0! | 0.22 | #DIV/0! | -0.09 | #DIV/0! | 0.13 | -0.09 |
| 9 | Afu3g13670 | siderochrome-iron transporter, putative | -0.30 | -0.16 | -0.02 | -0.30 | -0.23 | -0.08 | -0.16 | -0.20 |
| 9 | Afu3g13680 | conserved hypothetical protein | #DIV/0! | -0.80 | -0.18 | #DIV/0! | #DIV/0! | #DIV/0! | -0.49 |  |
| 9 | Afu3g13690 | pyoverdine chromophore biosynthetic protein, putative | 0.12 | 0.21 | 0.19 | 0.00 | 0.09 | -0.08 | 0.17 | 0.00 |
| 9 | Afu3g13700 | transferase family protein | -0.04 | 0.20 | -0.17 | -0.24 | 0.03 | -0.09 | 0.00 | -0.10 |
| 9 | Afu3g13710 | GTP cyclohydrolase I, putative | -0.04 | 0.09 | 0.12 | #DIV/0! | 0.14 | 0.15 | 0.06 | 0.14 |
| 9 | Afu3g13720 | oxidoreductase, 2OG-Fe(II) oxygenase family, putative | 0.08 | 0.01 | -0.15 | -0.13 | -0.14 | #DIV/0! | -0.02 | -0.13 |
| 9 | Afu3g13730 | **nonribosomal peptide synthase, putative** | 0.12 | 0.18 | 0.19 | 0.01 | -0.03 | 0.13 | 0.16 | 0.04 |
| 9 | Afu3g13740 | HSP70 family protein | -0.15 | 0.03 | -0.12 | 0.06 | -0.06 | #DIV/0! | -0.08 | 0.00 |
| 9 | **Afu3g13750** | conserved hypothetical protein | 0.37 | 0.43 | 0.08 | 0.19 | 0.28 | 0.18 | 0.29 | 0.21 |
|  |  |  |  |  |  |  |  |  |  |  |
| 10 | **Afu3g14560** | MFS multidrug transporter, putative | 0.64 | 0.58 | 0.39 | 0.24 | -0.03 | 0.35 | 0.54 | 0.19 |
| 10 | Afu3g14570 | acid phosphatase, putative | -0.10 | 0.41 | -0.15 | 0.11 | 0.13 | 0.24 | 0.05 | 0.16 |
| 10 | **Afu3g14580** | dUTPase, putative | 0.28 | 0.37 | 0.37 | 0.09 | 0.17 | -0.01 | 0.34 | 0.09 |
| 10 | Afu3g14590 | copper amine oxidase | 0.01 | -0.24 | 0.02 | -0.11 | -0.34 | 0.18 | -0.07 | -0.09 |
| 10 | **Afu3g14600** | fungal specific transcription factor, putative | 0.87 | 0.11 | -0.05 | 0.45 | 0.05 | -0.16 | 0.31 | 0.11 |
| 10 | **Afu3g14610** | cytochrome P450, putative | 1.65 | 1.28 | 1.42 | 0.61 | 0.78 | 0.60 | 1.45 | 0.66 |
| 10 | Afu3g14620 | extracellular endo-1,5-alpha-L-arabinase, putative | #DIV/0! | #DIV/0! | 0.07 | #DIV/0! | #DIV/0! | -0.18 | 0.07 | -0.18 |
| 10 | Afu3g14630 | extracellular dioxygenase, putative | -0.18 | -0.05 | -0.08 | -0.09 | -0.21 | -0.40 | -0.11 | -0.23 |
| 10 | Afu3g14640 | C4-dicarboxylate transporter/malic acid transport protein | #DIV/0! | #DIV/0! | #DIV/0! | #DIV/0! | 0.01 | #DIV/0! |  | 0.01 |
| 10 | Afu3g14650 | LipA domain protein, putative | -0.21 | -0.07 | -0.10 | 0.05 | -0.09 | 0.08 | -0.13 | 0.01 |
| 10 | Afu3g14660 | conserved hypothetical protein | -0.53 | #DIV/0! | -0.44 | -0.03 | -0.29 | 0.17 | -0.48 | -0.05 |
| 10 | Afu3g14670 | MFS transporter, putative | 0.03 | -0.14 | -0.17 | 0.22 | 0.55 | 0.27 | -0.09 | 0.35 |
| 10 | Afu3g14680 | lysophospholipase Plb3 | 0.25 | -0.26 | 0.30 | -0.21 | -0.99 | -0.23 | 0.10 | -0.48 |
| 10 | **Afu3g14690** | aminotransferase, putative | 0.22 | 0.25 | 0.28 | -0.13 | -0.18 | -0.01 | 0.25 | -0.11 |
| 10 | **Afu3g14700** | **polyketide synthase, putative** | 0.19 | 0.20 | 0.24 | -0.20 | -0.06 | -0.11 | 0.21 | -0.12 |
| 10 | **Afu3g14710** | toxin biosynthesis proten (Fum3), putative | 0.50 | 0.27 | 0.50 | -0.18 | -0.30 | -0.11 | 0.42 | -0.20 |
| 10 | **Afu3g14720** | MFS transporter, putative | 0.77 | 0.49 | 0.65 | -0.10 | -0.19 | -0.15 | 0.63 | -0.15 |
| 10 | **Afu3g14730** | LysR family regulatory protein, putative | 1.10 | 1.14 | 1.20 | -0.04 | 0.26 | 0.08 | 1.15 | 0.10 |
| 10 | **Afu3g14740** | conserved hypothetical protein | 1.45 | 1.28 | 1.51 | 0.21 | 0.06 | 0.31 | 1.41 | 0.19 |
| 10 | **Afu3g14750** | fungal specific transcription factor, putative | 0.69 | 0.68 | 0.68 | -0.26 | 0.21 | -0.08 | 0.68 | -0.04 |
| 10 | Afu3g14760 | cytochrome P450 monooxygenase (Fum15), putative | 0.00 | 0.03 | -0.07 | 0.03 | 0.29 | -0.02 | -0.01 | 0.10 |
|  |  |  |  |  |  |  |  |  |  |  |
| 11 | **Afu3g15200** | conserved hypothetical protein | 0.29 | 0.24 | 0.27 | -0.02 | 0.10 | -0.10 | 0.27 | -0.01 |
| 11 | Afu3g15210 | endo-1,4-beta-xylanase, putative | #DIV/0! | -0.31 | -0.41 | -0.16 | -0.15 | -0.06 | -0.36 | -0.12 |
| 11 | Afu3g15220 | conserved hypothetical protein | -0.41 | #DIV/0! | #DIV/0! | 0.11 | #DIV/0! | #DIV/0! | -0.41 | 0.11 |
| 11 | Afu3g15230 | general amidase, putative | -0.47 | -0.43 | -0.03 | 0.35 | -0.21 | 0.57 | -0.31 | 0.23 |
| 11 | Afu3g15240 | conserved hypothetical protein | #DIV/0! | #DIV/0! | -0.21 | #DIV/0! | -0.38 | #DIV/0! | -0.21 | -0.38 |
| 11 | Afu3g15250 | MFS toxin efflux pump, putative |  |  |  |  |  |  |  |  |
| 11 | Afu3g15260 | hypothetical protein | 0.16 | #DIV/0! | -0.24 | -0.20 | 0.10 | -0.19 | -0.04 | -0.10 |
| 11 | Afu3g15270 | **nonribosomal peptide synthetase, putative** | | |  |  |  |  |  |  |
| 11 | Afu3g15280 | methyltransferase, putative | -0.12 | 0.02 | -0.13 | -0.12 | -0.10 | 0.05 | -0.08 | -0.06 |
| 11 | Afu3g15290 | C6 transcription factor, putative | #DIV/0! | #DIV/0! | #DIV/0! | -0.09 | #DIV/0! | #DIV/0! |  | -0.09 |
| 11 | Afu3g15300 | amine transporter, putative | #DIV/0! | #DIV/0! | #DIV/0! | -0.02 | #DIV/0! | #DIV/0! |  | -0.02 |
| 11 | Afu3g15310 | **polyketide synthase, putative** | #DIV/0! | #DIV/0! | 0.01 | -0.36 | -0.05 | -0.06 |  | -0.16 |
| 11 | **Afu3g15330** | hypothetical protein | 0.18 | 0.49 | 0.60 | 0.37 | -0.04 | 0.56 | 0.42 | 0.30 |
| 11 | Afu3g15340 | hypothetical protein | 0.13 | 0.06 | 0.14 | -0.03 | 0.17 | 0.07 | 0.11 | 0.07 |
|  | Afu3g15350 | short chain dehydrogenase family protein, putative | -0.36 | -0.29 | 0.09 | 0.27 | -0.23 | #DIV/0! | -0.18 | 0.02 |
|  | **Afu3g15360** | LINE-1 calss reverse transcriptase, RNaseH, putative | | |  |  |  |  | 0.04 | -0.04 |
|  |  |  |  |  |  |  |  |  |  |  |
| 12 | **Afu4g00110** | TfdA family taurine dioxygenase, putative | 0.36 | 0.31 | 0.31 | 0.23 | 0.34 | 0.06 | 0.32 | 0.21 |
| 12 | Afu4g00120 | MFS transporter, putative |  |  |  |  |  |  |  |  |
| 12 | Afu4g00130 | hypothetical protein |  |  |  |  |  |  |  |  |
| 12 | Afu4g00140 | RTA1 domain protein, putative | -0.06 | -0.11 | -0.11 | #DIV/0! | #DIV/0! | #DIV/0! | -0.09 |  |
| 12 | Afu4g00150 | MFS maltose transporter, putative | -0.16 | -0.05 | -0.12 | -0.02 | -0.02 | 0.27 | -0.11 | 0.08 |
| 12 | Afu4g00160 | short-chain dehydrogenase, putative | -0.23 | -0.07 | -0.21 | -0.05 | -0.05 | 0.28 | -0.17 | 0.06 |
| 12 | Afu4g00170 | conserved hypothetical protein |  |  |  |  |  |  |  |  |
| 12 | Afu4g00180 | fatty acid oxygenase, putative | -0.01 | 0.13 | -0.02 | -0.01 | 0.01 | 0.02 | 0.04 | 0.01 |
| 12 | Afu4g00190 | conserved hypothetical protein |  |  |  |  |  |  |  |  |
| 12 | **Afu4g00200** | F-box domain protein | 0.84 | 0.78 | 0.84 | 0.56 | 0.29 | 0.67 | 0.82 | 0.51 |
| 12 | **Afu4g00210** | **polyketide synthase, putative** | 3.69 | 3.14 | 3.22 | 0.78 | 0.77 | 0.92 | 3.35 | 0.83 |
| 12 | **Afu4g00220** | metallo-beta-lactamase domain protein | 1.71 | 1.12 | 1.45 | 0.76 | 0.51 | 0.77 | 1.43 | 0.68 |
| 12 | **Afu4g00230** | oxidoreductase, 2OG-Fe(II) oxygenase family, putative | 2.90 | 2.10 | 2.75 | 0.80 | 0.54 | 0.96 | 2.58 | 0.76 |
| 12 | Afu4g00240 | amino acid transporter, putative | #DIV/0! | #DIV/0! | #DIV/0! | 0.33 | #DIV/0! | #DIV/0! |  | 0.33 |
| 12 | Afu4g00250 | WD40 repeat protein | 0.10 | -0.04 | 0.18 | 0.12 | 0.04 | 0.19 | 0.08 | 0.12 |
| 12 | Afu4g00260 | hypothetical protein |  |  |  |  |  |  |  |  |
| 12 | **Afu4g00270** | Fot5 family transposase, putative |  |  |  |  |  |  | 0.41 | 0.24 |
| 12 | **Afu4g00280** | conserved hypothetical protein | 3.44 | 3.06 | 3.38 | 0.40 | 0.00 | 0.68 | 3.29 | 0.36 |
|  |  |  |  |  |  |  |  |  |  |  |
| 13 | **Afu4g14370** | LINE-1 class reverse transcriptase, RNase H, putative | | |  |  |  |  | 0.56 | 0.37 |
| 13 | **Afu4g14380** | conserved hypothetical protein | 4.11 | 4.39 | 4.18 | 1.08 | 1.28 | 1.10 | 4.22 | 1.15 |
| 13 | **Afu4g14390** | Fot5 transposase, putative |  |  |  |  |  |  | 0.49 | 0.19 |
| 13 | **Afu4g14400** | C2H2 finger domain protein, putative | 0.43 | 0.09 | 0.38 | 0.07 | -0.15 | 0.23 | 0.30 | 0.05 |
| 13 | Afu4g14410 | hypothetical protein | -0.21 | #DIV/0! | -0.14 | #DIV/0! | #DIV/0! | #DIV/0! | -0.17 |  |
| 13 | Afu4g14420 | secreted glycosyl hydrolase, putative | -0.51 | #DIV/0! | -0.43 | -0.23 | -0.30 | -0.08 | -0.47 | -0.20 |
| 13 | Afu4g14440 | **NRPS-like enzyme, putative** | 0.06 | 0.14 | -0.05 | 0.10 | 0.17 | 0.15 | 0.05 | 0.14 |
| 13 | **Afu4g14450** | D-mannonate oxidoreductase | 1.13 | 1.42 | 1.09 | -0.05 | 0.22 | -0.19 | 1.21 | -0.01 |
| 13 | Afu4g14460 | conserved hypothetical protein |  |  |  |  |  |  |  |  |
| 13 | Afu4g14470 | hypothetical protein |  |  |  |  |  |  |  |  |
| 13 | Afu4g14480 | conserved hypothetical protein | #DIV/0! | #DIV/0! | -0.17 | #DIV/0! | #DIV/0! | 0.02 | -0.17 | 0.02 |
| 13 | Afu4g14490 | extracellular dihydrogeodin oxidase/laccase, putative | | |  |  |  |  |  |  |
| 13 | Afu4g14500 | conserved hypothetical protein |  |  |  |  |  |  |  |  |
| 13 | Afu4g14510 | hypothetical protein | #DIV/0! | 0.02 | #DIV/0! | 0.03 | 0.04 | -0.16 | 0.02 | -0.03 |
| 13 | **Afu4g14520** | toxin biosynthesis protein, putative | 0.49 | 0.25 | 0.28 | 0.12 | 0.29 | 0.03 | 0.34 | 0.15 |
| 13 | **Afu4g14530** | glutathione S-transferase, putative | 3.85 | 2.99 | 3.56 | 0.71 | 0.37 | 0.90 | 3.47 | 0.66 |
| 13 | **Afu4g14540** | C6 transcription factor (AflR), putative | 1.84 | 0.74 | 1.48 | 0.58 | 0.11 | 0.58 | 1.36 | 0.42 |
| 13 | Afu4g14550 | toxin biosynthesis regulatory protein AFLJ, putative | | |  |  |  |  |  |  |
| 13 | Afu4g14560 | **polyketide synthase, putative** |  |  |  |  |  |  |  |  |
| 13 | Afu4g14570 | metallo-beta-lactamase domain protein, putative | 0.27 | 0.03 | 0.06 | 0.21 | -0.05 | 0.48 | 0.12 | 0.21 |
| 13 | Afu4g14580 | O-methyltransferase, putative | #DIV/0! | 0.12 | -0.03 | -0.01 | -0.01 | -0.17 | 0.04 | -0.06 |
| 13 | Afu4g14590 | C6 transcription factor, putative | -0.03 | 0.15 | -0.20 | -0.13 | -0.31 | 0.10 | -0.03 | -0.12 |
| 13 | Afu4g14600 | UPF0075 domain protein |  |  |  |  |  |  |  |  |
| 13 | Afu4g14610 | MFS sugar transporter, putative | -0.03 | 0.01 | -0.02 | 0.13 | 0.21 | -0.01 | -0.02 | 0.11 |
| 13 | Afu4g14620 | RING finger protein |  |  |  |  |  |  |  |  |
| 13 | Afu4g14630 | FAD binding oxidoreductase, putative | |  |  |  |  |  |  |  |
| 13 | Afu4g14640 | low affinity iron transporter, putative | |  |  |  |  |  |  |  |
| 13 | Afu4g14650 | ribokinase | -0.34 | -0.42 | -0.45 | -0.01 | 0.35 | -0.19 | -0.40 | 0.05 |
| 13 | Afu4g14660 | conserved hypothetical protein | -0.20 | #DIV/0! | #DIV/0! | -0.08 | 0.06 | #DIV/0! | -0.20 | -0.01 |
| 13 | Afu4g14670 | MFS quinate transporter, putative |  |  |  |  |  |  |  |  |
| 13 | **Afu4g14680** | C6 transcription factor, putative | -0.30 | -0.37 | -0.03 | 0.06 | 0.08 | -0.29 | -0.23 | -0.05 |
| 13 | Afu4g14690 | hypothetical protein | 0.04 | #DIV/0! | 0.05 | #DIV/0! | #DIV/0! | #DIV/0! | 0.05 |  |
| 13 | Afu4g14700 | GDSL-like Lipase/Acylhydrolase family protein | | |  |  |  |  |  |  |
| 13 | Afu4g14710 | MFS transporter, putative |  |  |  |  |  |  |  |  |
| 13 | Afu4g14720 | C6 transcription factor, putative |  |  |  |  |  |  | 0.04 | -0.04 |
| 13 | Afu4g14730 | GNAT family acetyltransferase, putative | 0.01 | -0.44 | #DIV/0! | -0.09 | 0.12 | 0.01 | -0.21 | 0.01 |
| 13 | Afu4g14740 | serine/threonine protein kinase (Ark1), putative | 0.70 | #DIV/0! | 0.81 | 0.07 | 0.08 | 0.11 | 0.76 | 0.09 |
| 13 | Afu4g14750 | hypothetical protein |  |  |  |  |  |  |  |  |
| 13 | Afu4g14760 | ABC multidrug transporter, putative | |  |  |  |  |  |  |  |
| 13 | **Afu4g14770** | squalene-hopene-cyclase, putative | 3.65 | 3.57 | 3.09 | 0.30 | 0.72 | 0.17 | 3.43 | 0.40 |
| 13 | **Afu4g14780** | cytochrome P450 monooxygenase, putative | 3.34 | 2.26 | 2.54 | 0.36 | -0.01 | 0.42 | 2.71 | 0.26 |
| 13 | **Afu4g14790** | cytochrome P450 monooxygenase, putative | 3.67 | 2.80 | 2.95 | 0.46 | 0.71 | 0.36 | 3.14 | 0.51 |
| 13 | **Afu4g14800** | short chain dehydrogenase, putative | 3.36 | 2.68 | 2.81 | 0.30 | 0.22 | 0.46 | 2.95 | 0.33 |
| 13 | **Afu4g14810** | cytochrome P450 monooxygenase, putative | 1.92 | 1.32 | 1.91 | 0.42 | 0.28 | 0.35 | 1.72 | 0.35 |
| 13 | **Afu4g14820** | transferase family protein | 1.64 | 1.50 | 1.45 | 0.15 | 0.47 | 0.10 | 1.53 | 0.24 |
| 13 | **Afu4g14830** | cytochrome P450 monooxygenase, putative | 2.11 | 1.67 | 1.41 | 0.43 | 0.20 | 0.29 | 1.73 | 0.30 |
| 13 | **Afu4g14840** | transferase family protein | 2.01 | 0.52 | 1.35 | 0.51 | -0.43 | 0.59 | 1.29 | 0.22 |
| 13 | **Afu4g14850** | extracellular 3-ketosteroid 1-dehydrogenase, putative | 1.72 | 1.77 | 1.40 | 0.35 | 0.73 | 0.29 | 1.63 | 0.46 |
| 13 | Afu4g14870 | LINE-1 class reverse transcriptase, RNaseH, putative | | |  |  |  |  | 0.19 | 0.19 |
| 13 | **Afu4g14880** | reverse transcriptase, RNaseH, putative | |  |  |  |  |  | 0.71 | 0.58 |
|  |  |  |  |  |  |  |  |  |  |  |
|  | Afu5g00100 | integral membrane protein | -0.26 | -0.02 | #DIV/0! | #DIV/0! | #DIV/0! | 0.01 | -0.14 |  |
| 14 | Afu5g00110 | squalene-hopene-cyclase, putative | -0.13 | -0.26 | -0.19 | 0.10 | 0.25 | -0.01 | -0.19 | 0.12 |
| 14 | Afu5g00120 | cytochrome P450 oxidoreductase/alkane hydroxylase, putative | 0.09 | 0.14 | 0.22 | 0.04 | -0.09 | 0.05 | 0.15 | 0.00 |
| 14 | Afu5g00130 | capsule polysaccharide biosynthesis protein, putative | 0.23 | -0.10 | -0.15 | 0.01 | 0.21 | 0.12 | -0.01 | 0.11 |
| 14 | Afu5g00140 | transposase, putative |  |  |  |  |  |  |  |  |
| 14 | Afu5g00150 | **nonribosomal peptide synthetase, putative** | -0.15 | 0.27 | -0.27 | -0.34 | 0.27 | -0.32 | -0.05 | -0.13 |
| 14 | Afu5g00160 | MFS polyamine transporter, putative | #DIV/0! | #DIV/0! | #DIV/0! | -0.21 | #DIV/0! | #DIV/0! |  | -0.21 |
|  |  |  |  |  |  |  |  |  |  |  |
| 15 | Afu5g00340 | ABC multidrug transporter, putative | -0.16 | -0.05 | 0.03 | -0.01 | 0.17 | 0.11 | -0.06 | 0.09 |
| 15 | Afu5g00350 | **nonribosomal peptide synthetase, putative** | -0.02 | #DIV/0! | 0.00 | -0.09 | 0.19 | #DIV/0! | -0.02 | 0.05 |
| 15 | Afu5g00370 | amino acid permease, putative |  |  |  |  |  |  |  |  |
| 15 | Afu5g00380 | TPR domain protein | -0.38 | #DIV/0! | #DIV/0! | -0.13 | 0.11 | #DIV/0! | -0.38 | -0.01 |
| 15 | Afu5g00390 | hypothetical protein | #DIV/0! | #DIV/0! | 0.36 | 0.30 | -0.24 | #DIV/0! | 0.36 | 0.03 |
| 15 | Afu5g00400 | aminotransferase, classes I and II family, putative | | |  |  |  |  |  |  |
|  |  |  |  |  |  |  |  |  |  |  |
| 16 | Afu5g12700 | hypothetical protein | -0.22 | -0.22 | -0.16 | -0.04 | -0.16 | 0.09 | -0.20 | -0.03 |
| 16 | Afu5g12710 | TPR domain protein |  |  |  |  |  |  |  |  |
| 16 | Afu5g12720 | ABC multidrug transporter, putative | 0.23 | -0.07 | 0.17 | 0.35 | 0.04 | 0.49 | 0.11 | 0.30 |
| 16 | Afu5g12730 | **nonribosomal peptide synthase, putative** | 0.13 | 0.11 | 0.29 | 0.18 | -0.25 | 0.22 | 0.17 | 0.05 |
| 16 | Afu5g12740 | MFS drug transporter, putative | 0.14 | #DIV/0! | -0.16 | 0.18 | #DIV/0! | 0.47 |  | 0.32 |
|  |  |  |  |  |  |  |  |  |  |  |
| 17 | **Afu6g03290** | isochorismatase family hydrolase, putative | 1.06 | 1.43 | 1.41 | 0.61 | 0.77 | 0.69 | 1.30 | 0.69 |
| 17 | Afu6g03300 | conserved hypothetical protein | #DIV/0! | 0.11 | #DIV/0! | 0.28 | -0.03 | #DIV/0! | 0.11 | 0.13 |
| 17 | **Afu6g03310** | conserved hypothetical protein | 0.43 | 0.35 | 0.38 | 0.27 | 0.11 | 0.48 | 0.39 | 0.29 |
| 17 | **Afu6g03320** | MFS drug transporter, putative | 0.25 | 0.32 | 0.20 | -0.09 | -0.17 | -0.10 | 0.26 | -0.12 |
| 17 | Afu6g03330 | conserved hypothetical protein | -0.01 | -0.04 | #DIV/0! | -0.25 | -0.07 | #DIV/0! | -0.03 | -0.16 |
| 17 | Afu6g03340 | conserved hypothetical protein | -1.12 | -1.39 | -0.82 | #DIV/0! | #DIV/0! | #DIV/0! | -1.11 |  |
| 17 | Afu6g03350 | acetyltransferase, GNAT family, putative | -1.10 | -0.93 | -0.79 | -0.16 | -0.05 | 0.03 | -0.94 | -0.06 |
| 17 | Afu6g03360 | ToxD-like zinc binding oxidoreductase, putative | -0.10 | 0.00 | -0.14 | -0.08 | 0.32 | 0.07 | -0.08 | 0.10 |
| 17 | Afu6g03370 | oxidoreductase, short-chain dehydrogenase/reductase family | | | |  |  |  | 0.22 | -0.20 |
| 17 | **Afu6g03380** | conserved hypothetical protein | 0.22 | 0.10 | 0.34 | -0.21 | -0.40 | 0.00 | -0.13 | 0.07 |
| 17 | Afu6g03390 | glutathione S-transferase family protein | #DIV/0! | -0.13 | #DIV/0! | 0.07 | #DIV/0! | #DIV/0! | 0.93 | 0.26 |
| 17 | **Afu6g03400** | hypothetical protein | 1.04 | 0.42 | 1.34 | 0.64 | -0.74 | 0.89 | 1.19 | 0.36 |
| 17 | **Afu6g03420** | trehalose synthase, putative |  |  |  |  |  |  | 1.11 | 0.28 |
| 17 | **Afu6g03430** | C6 finger domain protein, putative | 3.00 | 2.54 | 1.99 | -0.37 | -0.10 | -0.35 | 2.51 | -0.27 |
| 17 | **Afu6g03440** | fructosyl amino acid oxidase, putative | 3.83 | 4.17 | 3.45 | -1.09 | -0.34 | -1.16 | 3.82 | -0.86 |
| 17 | **Afu6g03450** | conserved hypothetical protein | 1.83 | 1.42 | 1.43 | -0.66 | -0.89 | -0.50 | 1.56 | -0.68 |
| 17 | **Afu6g03460** | conserved hypothetical protein | 2.57 | 2.51 | 2.24 | -0.35 | -0.31 | -0.34 | 2.44 | -0.33 |
| 17 | **Afu6g03470** | ABC multidrug transporter, putative | 1.43 | 1.56 | 1.55 | -0.61 | -0.58 | -0.56 | 1.51 | -0.58 |
| 17 | **Afu6g03480** | **nonribosomal peptide synthetase, putative** | 1.07 | 0.41 | 0.72 | -0.21 | -0.81 | -0.13 | 0.73 | -0.38 |
| 17 | **Afu6g03490** | phenol 2-monooxygenase, putative | 1.30 | 0.66 | 1.09 | -0.55 | -0.69 | -0.22 | 1.02 | -0.49 |
|  |  |  |  |  |  |  |  |  |  |  |
|  |  | **Gliotoxin cluster** |  |  |  |  |  |  |  |  |
| 18 | **Afu6g09580** | conserved hypothetical protein | 0.65 | 0.14 | 0.30 | 0.04 | 0.14 | -0.07 | 0.36 | 0.04 |
| 18 | **Afu6g09590** | zinc alcohol dehydrogenase, putative | 0.96 | #DIV/0! | #DIV/0! | 0.53 | 0.12 | 0.69 | 0.96 | 0.45 |
| 18 | **Afu6g09600** | zinc metallopeptidase, putative | 2.34 | 2.18 | 1.91 | 0.53 | 0.96 | 0.20 | 2.14 | 0.56 |
| 18 | **Afu6g09610** | **nonribosomal peptide synthase, putative** | 3.25 | 2.32 | 2.90 | -0.18 | 0.00 | -0.13 | 2.82 | -0.11 |
| 18 | **Afu6g09620** | hypothetical protein | 1.00 | 1.00 | 0.90 | -0.02 | -0.05 | 0.22 | 0.97 | 0.05 |
| 18 | **Afu6g09630** | C6 finger domain protein GliZ | 2.35 | 1.09 | 1.61 | 0.10 | -0.08 | -0.02 | 1.68 | 0.00 |
| 18 | **Afu6g09640** | aminotransferase GliI | 2.93 | 1.59 | 2.44 | -0.38 | -1.05 | -0.32 | 2.32 | -0.59 |
| 18 | **Afu6g09650** | membrane dipeptidase GliJ | 3.04 | 1.98 | 2.42 | -0.21 | -0.10 | -0.23 | 2.48 | -0.18 |
| 18 | **Afu6g09660** | **nonribosomal peptide synthase GliP** | 3.42 | 2.45 | 3.35 | -0.24 | -1.01 | -0.04 | 3.07 | -0.43 |
| 18 | **Afu6g09670** | cytochrome P450 oxidoreductase GliC | 4.55 | 3.34 | 3.68 | -0.23 | -0.53 | 0.01 | 3.86 | -0.25 |
| 18 | **Afu6g09680** | O-methyltransferase GliM | 5.22 | 3.17 | 3.96 | 0.13 | -0.99 | 0.30 | 4.12 | -0.19 |
| 18 | **Afu6g09690** | glutathione S-transferase GliG | 5.15 | 4.82 | 4.89 | -0.31 | -0.82 | -0.04 | 4.95 | -0.39 |
| 18 | **Afu6g09700** | gliotoxin biosynthesis protein GliK | 4.50 | 2.79 | 3.79 | 0.00 | -0.65 | 0.34 | 3.69 | -0.10 |
| 18 | **Afu6g09710** | MFS gliotoxin efflux pump GliA | 3.50 | 2.52 | 3.14 | 0.25 | -0.79 | 0.32 | 3.05 | -0.07 |
| 18 | **Afu6g09720** | methyltransferase GliN | 4.51 | 3.31 | 3.61 | 0.18 | -0.73 | 0.37 | 3.81 | -0.06 |
| 18 | **Afu6g09730** | cytochrome P450 oxidoreductase GliF | 4.75 | 4.68 | 4.50 | -0.28 | -0.13 | -0.32 | 4.64 | -0.25 |
| 18 | **Afu6g09740** | thioredoxin reductase GliT | 4.70 | 4.43 | 4.59 | 0.09 | -0.46 | 0.12 | 4.57 | -0.08 |
| 18 | Afu6g09750 | retrotransposon, pseudogene |  |  |  |  |  |  | -0.04 |  |
| 18 | Afu6g09760 | toxin biosynthesis cytochrome P450 oxidoreductase, putative | #DIV/0! | #DIV/0! | #DIV/0! | 0.21 | #DIV/0! | #DIV/0! |  |  |
| 18 | **Afu6g09770** | geranylgeranyl diphosphate synthase, putative | 0.91 | 0.50 | 0.68 | 0.22 | 0.49 | 0.05 | 0.70 | 0.25 |
|  |  |  |  |  |  |  |  |  |  |  |
| 19 | **Afu6g12040** | MFS sugar permease, putative | 0.40 | 0.94 | 0.67 | 0.05 | 0.45 | 0.16 | 0.67 | 0.22 |
| 19 | **Afu6g12050** | **nonribosomal peptide synthase, putative** | 3.67 | 3.43 | 3.48 | 0.81 | 0.71 | 0.79 | 3.53 | 0.77 |
| 19 | **Afu6g12060** | MAK1-like monooxygenase, putative | 2.51 | 2.45 | 3.15 | 1.19 | 0.62 | 1.18 | 2.70 | 1.00 |
| 19 | **Afu6g12070** | FAD binding domain protein | 5.05 | 5.15 | 4.93 | 0.90 | 1.27 | 0.96 | 5.04 | 1.04 |
| 19 | Afu6g12080 | **nonribosomal peptide synthase, putative** | | |  |  |  |  | 3.60 | 0.83 |
|  |  |  |  |  |  |  |  |  |  |  |
| 20 | **Afu6g13920** | adenylate-forming enzyme, putative | 3.97 | 3.40 | 3.43 | 0.86 | 0.85 | 0.79 | 0.78 | 0.57 |
| 20 | **Afu6g13930** | **polyketide synthase, putative** | 0.64 | #DIV/0! | 0.93 | 0.57 | 0.41 | #DIV/0! | 2.17 | 0.53 |
| 20 | **Afu6g13940** | cytochrome P450 monooxygenase, putative | 2.50 | 2.18 | 1.83 | 0.40 | 0.96 | 0.24 | 1.23 | 0.36 |
| 20 | **Afu6g13950** | integral membrane protein | 1.39 | 1.06 | #DIV/0! | 0.56 | 0.34 | 0.18 | 1.21 | 0.64 |
| 20 | **Afu6g13970** | FAD-dependent monooxygenase (PaxM), putative | 2.19 | 2.05 | 2.06 | 0.58 | 0.33 | 0.75 | 2.10 | 0.55 |
| 20 | **Afu6g13980** | UbiA-like prenyltransferase, putative | 2.36 | 2.41 | 2.10 | 0.77 | 0.23 | 0.75 | 2.29 | 0.58 |
| 20 | **Afu6g13990** | acetyltransferase, putative | 1.20 | 1.12 | 1.13 | 0.50 | 0.49 | 0.35 | 1.15 | 0.45 |
| 20 | **Afu6g14000** | Tri7-like toxin biosynthesis protein, putative | 0.29 | 0.24 | 0.20 | 0.37 | -0.23 | 0.28 | 0.24 | 0.14 |
|  |  |  |  |  |  |  |  |  |  |  |
| 21 | **Afu7g00100** | LINE-1 class reverse transcriptase/RNaseH, pseudogene | | | |  |  |  | 0.50 | 0.43 |
| 21 | Afu7g00110 | secretory lipase, putative | #DIV/0! | -0.06 | -0.09 | -0.21 | 0.08 | #DIV/0! | -0.07 | -0.06 |
| 21 | Afu7g00120 | metallo-beta-lactamase domain protein | -0.16 | -0.03 | 0.00 | #DIV/0! | 0.17 | #DIV/0! | -0.06 | 0.17 |
| 21 | Afu7g00130 | C6 transcription factor, putative |  |  |  |  |  |  |  |  |
| 21 | Afu7g00150 | FAD-dependent monooxygenase, putative | 0.18 | 0.49 | -0.17 | -0.04 | 0.49 | -0.20 | 0.17 | 0.09 |
| 21 | Afu7g00160 | **polyketide synthase, putative** | #DIV/0! | -0.06 | -0.34 | -0.28 | 0.20 | 0.10 | -0.20 | 0.01 |
| 21 | Afu7g00170 | **dimethylallyl tryptophan synthase (GliD), putative** | -0.15 | -0.05 | -0.43 | -0.13 | 0.49 | -0.31 | -0.21 | 0.02 |
| 21 | **Afu7g00180** | NAD dependent epimerase/dehydratase, putative | 0.64 | 0.50 | 0.47 | 0.02 | 0.08 | 0.17 | 0.54 | 0.09 |
| 21 | Afu7g00190 | hypothetical protein |  |  |  |  |  |  |  |  |
|  |  |  |  |  |  |  |  |  |  |  |
|  |  | **fumitremorgin B and other products** | |  |  |  |  |  |  |  |
| 22 | **Afu8g00100** | aspartate-tRNA ligase, putative | 1.72 | 1.46 | 1.03 | 0.55 | 0.80 | 0.26 | 1.40 | 0.54 |
| 22 | **Afu8g00110** | oxidoreductase, 2OG-Fe(II) oxygenase family, putative | 0.71 | 0.39 | 0.84 | 0.24 | 0.49 | 0.53 | 0.65 | 0.42 |
| 22 | Afu8g00120 | hypothetical protein | #DIV/0! | #DIV/0! | #DIV/0! | 0.02 | 0.32 | #DIV/0! |  | 0.17 |
| 22 | **Afu8g00130** | hypothetical protein | 1.58 | 1.37 | 1.27 | 0.43 | 0.77 | 0.49 | 1.41 | 0.57 |
| 22 | Afu8g00140 | MFS transporter, putative |  |  |  |  |  |  |  |  |
| 22 | Afu8g00150 | conserved hypothetical protein |  |  |  |  |  |  |  |  |
| 22 | **Afu8g00160** | conserved hypothetical protein | 1.21 | 0.72 | 0.78 | 0.26 | 0.19 | 0.16 | 0.91 | 0.20 |
| 22 | **Afu8g00170** | **nonribosomal peptide synthetase, putative** | 3.89 | 2.40 | 3.17 | 0.56 | -0.16 | 0.63 | 3.15 | 0.34 |
| 22 | **Afu8g00190** | cytochrome P450, putative | 3.34 | 3.11 | 2.93 | 0.45 | 0.44 | 0.31 | 3.13 | 0.40 |
| 22 | **Afu8g00200** | O-methyltransferase, putative | 2.81 | 2.11 | 2.41 | 0.45 | 0.35 | 0.53 | 2.44 | 0.44 |
| 22 | **Afu8g00210** | **dimethylallyl tryptophan synthase, putative** | 5.14 | 3.97 | 4.22 | 0.50 | 0.16 | 0.54 | 4.44 | 0.40 |
| 22 | **Afu8g00220** | cytochrome P450, putative | 2.06 | 1.13 | 1.73 | 0.29 | 0.07 | 0.41 | 1.64 | 0.25 |
| 22 | **Afu8g00230** | phytanoyl-CoA dioxygenase family protein | 3.32 | 2.27 | 2.59 | 0.29 | 0.00 | 0.39 | 2.73 | 0.23 |
| 22 | **Afu8g00240** | cytochrome P450 monooxygenase, putative | 3.14 | 1.98 | 2.65 | 0.32 | 0.17 | 0.17 | 2.59 | 0.22 |
| 22 | **Afu8g00250** | **dimethylallyl tryptophan synthase, putative** | 4.01 | 3.53 | 3.47 | 0.48 | 0.70 | 0.50 | 3.67 | 0.56 |
| 22 | **Afu8g00260** | F-box domain and ankyrin repeat protein | 1.12 | 0.53 | 1.12 | 0.11 | -0.03 | 0.20 | 0.92 | 0.09 |
| 22 | Afu8g00270 | **polyketide synthase, putative** |  |  |  |  |  |  |  |  |
| 22 | Afu8g00280 | short-chain dehydrogenase, putative | #DIV/0! | #DIV/0! | #DIV/0! | 0.13 | 0.15 | #DIV/0! |  | 0.14 |
| 22 | **Afu8g00290** | conserved hypothetical protein | 0.63 | 0.72 | 0.35 | 0.12 | 0.27 | -0.09 | 0.56 | 0.10 |
| 22 | **Afu8g00300** | conserved hypothetical protein | 0.29 | 0.64 | 0.44 | 0.04 | 0.33 | 0.14 | 0.45 | 0.17 |
| 22 | **Afu8g00310** | LINE-1 class reverse transcriptase, RNaseH, putative | | |  |  |  |  | 0.06 | 0.21 |
| 22 | **Afu8g00330** | Afut1endonuclease/reverse transcriptase/RNaseH, putative | | | |  |  |  | 0.23 | 0.20 |
| 22 | **Afu8g00340** | conserved hypothetical protein | 0.96 | #DIV/0! | 0.88 | 0.15 | 0.23 | -0.33 | 0.92 | 0.02 |
| 22 | **Afu8g00350** | conserved hypothetical protein | 1.16 | #DIV/0! | 1.02 | -0.03 | 0.02 | -0.39 | 1.09 | -0.13 |
| 22 | **Afu8g00360** | NlpC/P60-like cell-wall peptidase, putative | 1.62 | 1.31 | 1.32 | 0.14 | 0.14 | 0.09 | 1.42 | 0.12 |
| 22 | **Afu8g00370** | **polyketide synthase, putative** | 4.48 | 4.34 | 4.24 | -0.23 | 0.11 | -0.21 | 4.35 | -0.11 |
| 22 | **Afu8g00380** | DltD N-terminal domain protein | 1.02 | 0.90 | 1.03 | -0.16 | -0.21 | -0.08 | 0.98 | -0.15 |
| 22 | **Afu8g00390** | O-methyltransferase, putative | 5.31 | 4.33 | 4.88 | -0.33 | -0.80 | -0.05 | 4.84 | -0.39 |
| 22 | **Afu8g00400** | conserved hypothetical protein | 3.82 | 3.53 | 2.93 | -0.30 | -0.06 | -0.33 | 3.43 | -0.23 |
| 22 | **Afu8g00410** | methionine aminopeptidase, type II, putative | 3.05 | 2.82 | 2.67 | 0.07 | 0.13 | 0.23 | 2.85 | 0.14 |
| 22 | **Afu8g00420** | C6 finger transcription factor, putative | 4.21 | 3.17 | 3.88 | 0.07 | 0.11 | 0.14 | 3.75 | 0.11 |
| 22 | **Afu8g00430** | conserved hypothetical protein | 5.34 | 5.15 | 4.76 | -0.07 | 0.07 | -0.01 | 5.08 | 0.00 |
| 22 | **Afu8g00440** | steroid monooxygenase, putative | 4.72 | 4.17 | 3.99 | 0.12 | 0.43 | 0.18 | 4.29 | 0.24 |
| 22 | **Afu8g00460** | methionine aminopeptidase, type I, putative | 1.06 | 1.46 | 0.90 | 0.01 | 0.70 | 0.02 | 1.14 | 0.24 |
| 22 | **Afu8g00470** | conserved hypothetical protein | 1.03 | 1.11 | 0.93 | -0.01 | 0.29 | -0.03 | 1.02 | 0.08 |
| 22 | **Afu8g00480** | phytanoyl-CoA dioxygenase family protein | 4.90 | 4.41 | 4.69 | 0.34 | 0.65 | 0.46 | 4.67 | 0.48 |
| 22 | **Afu8g00490** | **polyketide synthase, putative** | 3.02 | 2.80 | 2.40 | 0.16 | 0.21 | 0.18 | 2.74 | 0.18 |
| 22 | **Afu8g00500** | acetate-CoA ligase, putative | 4.14 | 4.01 | 3.78 | 0.00 | 0.28 | 0.02 | 3.98 | 0.10 |
| 22 | **Afu8g00510** | O-methylsterigmatocystin oxidoreductase, putative | 3.61 | 3.17 | 3.51 | -0.18 | -0.21 | -0.05 | 3.43 | -0.15 |
| 22 | **Afu8g00520** | integral membrane protein | 3.77 | 3.63 | 3.19 | -0.13 | -0.08 | -0.03 | 3.53 | -0.08 |
| 22 | **Afu8g00530** | alpha/beta hydrolase, putative | 2.28 | 2.27 | 2.11 | 0.09 | 0.26 | 0.11 | 2.22 | 0.16 |
| 22 | **Afu8g00540** | **hybrid polyketide synthase/nonribosomal peptide synthase, putative** | 5.40 | 4.88 | 4.52 | -0.05 | -0.12 | 0.07 | 4.93 | -0.03 |
| 22 | **Afu8g00550** | methyltransferase SirN-like, putative | 5.82 | 5.96 | 5.79 | 0.15 | 0.26 | 0.19 | 5.86 | 0.20 |
| 22 | **Afu8g00560** | cytochrome P450, putative | 4.98 | 4.50 | 4.19 | 0.09 | 0.30 | 0.15 | 4.56 | 0.18 |
| 22 | **Afu8g00570** | alpha/beta hydrolase, putative | 2.89 | 1.84 | 2.53 | 0.18 | -0.49 | 0.18 | 2.42 | -0.04 |
| 22 | **Afu8g00580** | translation elongation factor eEF-1, gamma subunit, putative | 5.71 | 5.54 | 5.24 | -0.08 | 0.63 | -0.05 | 5.50 | 0.17 |
| 22 | **Afu8g00590** | fatty acid desaturase, putative | 0.29 | 0.00 | 0.35 | 0.27 | 0.00 | 0.26 | 0.21 | 0.18 |
| 22 | Afu8g00600 | conserved hypothetical protein |  |  |  |  |  |  |  |  |
| 22 | Afu8g00610 | cell surface protein Mas1, putative |  |  |  |  |  |  |  |  |
| 22 | Afu8g00620 | **dimethylallyl tryptophan synthase, putative** | #DIV/0! | 0.72 | 0.28 | 0.31 | 0.85 | 0.16 | 0.50 | 0.44 |
| 22 | **Afu8g00630** | conserved hypothetical protein | 2.17 | 1.42 | 1.32 | 0.79 | 0.92 | 0.60 | 1.64 | 0.77 |
| 22 | **Afu8g00640** | glycosyl transferase, putative | 1.78 | 1.46 | 1.38 | 0.63 | 0.80 | 0.65 | 1.54 | 0.69 |
| 22 | Afu8g00650 | LPS glycosyltransferase, putative | 0.16 | -0.69 | 0.19 | 0.15 | -0.74 | 0.25 | -0.11 | -0.12 |
| 22 | Afu8g00660 | solute transporter, putative |  |  |  |  |  |  |  |  |
| 22 | **Afu8g00670** | integral membrane protein | 0.38 | 0.47 | -0.12 | 0.20 | 0.08 | 0.06 | 0.24 | 0.11 |
| 22 | **Afu8g00680** | glycosyl transferase, group 2 family protein | 0.71 | #DIV/0! | 0.46 | 0.00 | #DIV/0! | -0.03 | 0.59 | -0.01 |
| 22 | Afu8g00690 | hypothetical protein | #DIV/0! | #DIV/0! | #DIV/0! | #DIV/0! | -0.10 | #DIV/0! |  | -0.10 |
| 22 | Afu8g00700 | class III chitinase ChiA2 | 0.29 | 0.13 | #DIV/0! | -0.07 | 0.73 | -0.13 | 0.21 | 0.18 |
| 22 | **Afu8g00710** | secreted antimicrobial peptide, putative | 2.08 | 1.74 | 1.70 | 0.81 | 0.40 | 0.72 | 1.84 | 0.64 |
| 22 | **Afu8g00720** | amino acid transporter, putative | 1.60 | 1.24 | 1.24 | 0.50 | 0.55 | 0.20 | 1.36 | 0.42 |
